# Macrophage piezo1 senses mechanical force to drive osteoclastogenesis via ZBP1: Implications for bone remodelling therapy

**DOI:** 10.1002/ctm2.70703

**Published:** 2026-05-23

**Authors:** Linlin Zhu, Kejia Zhang, An Song, Weiye Zhao, Yongze Lin, Hao Xu, Zhichun Jin, Yan Wang, Shengnan Wu, Xueyu Sun, Heng Gu, Ming Lu, Hanwen Zhang, Bin Yan

**Affiliations:** ^1^ Department of Orthodontics The Affiliated Stomatological Hospital of Nanjing Medical University Nanjing Jiangsu China; ^2^ State Key Laboratory Cultivation Base of Research Prevention and Treatment for Oral Diseases Nanjing Medical University Nanjing Jiangsu China; ^3^ Jiangsu Province Engineering Research Center of Stomatological Translational Medicine Nanjing Jiangsu China; ^4^ Department of Oral and Maxillofacial Surgery The Affiliated Stomatological Hospital of Nanjing Medical University Nanjing Jiangsu China; ^5^ Department of Stomatology The Affiliated Suzhou Hospital of Nanjing Medical University Suzhou Municipal Hospital Suzhou Jiangsu China; ^6^ Jiangsu Key Laboratory of Neurodegeneration Department of Pharmacology Nanjing Medical University Nanjing Jiangsu China; ^7^ Key Laboratory of Targeted Intervention of Cardiovascular Disease Collaborative Innovation Center for Cardiovascular Disease Translational Medicine Nanjing Medical University Nanjing Jiangsu China

**Keywords:** macrophage, mechanotransduction, orthodontic tooth movement, osteoclastogenesis, Piezo1, ZBP1

## Abstract

**Background:**

The unpredictability of orthodontic tooth movement (OTM) and risks of root resorption stem from poorly understood mechanisms governing alveolar bone remodelling. Specifically, how macrophages transduce mechanical forces into osteoclastogenic signals remains elusive.

**Methods:**

Our integrated approach combined analyses at multiple levels: clinical analysis of human periodontal ligament (PDL) tissues via qPCR and immunofluorescence; in vivo functional assessment using murine OTM and fracture models with macrophage‐specific knockouts (for *Piezo1* and Z‐DNA binding protein 1 (*Zbp1*), designated as *Piezo1^cko^* and *Zbp1^cko^*); and in vitro mechanistic investigation through RNA sequencing of murine bone marrow‐derived macrophages (BMDMs) and pharmacological screening. This multi‐faceted strategy was employed to dissect the pathway.

**Results:**

Mechanical stress activated macrophage Piezo1, triggering Ca^2+^ influx and subsequent upregulation of ZBP1. This axis was essential for pro‐inflammatory cytokine release and osteoclastogenesis. Macrophage‐specific deletion of *Piezo1* or *Zbp1* significantly decelerated OTM and preserved alveolar bone mass. Notably, *Zbp1* overexpression rescued the remodelling defects in *Piezo1*‐deficient mice. Virtual screening identified JNJ‐10311795 as a ZBP1 modulator; its administration effectively slowed OTM and, conversely, accelerated fracture healing by shifting the balance towards bone formation.

**Conclusion:**

The Piezo1–ZBP1 axis constitutes a novel mechano‐immune switch converting physical stress into inflammation and bone resorption. Pharmacological targeting of this axis offers a bidirectional therapeutic strategy for controlling orthodontic movement and enhancing bone repair.

## Key points

- Macrophage Piezo1 is a key mechanosensor in OTM and fracture healing.
- ZBP1 is identified as a novel downstream effector of Piezo1 in sterile mechanotransduction.
- The Piezo1–ZBP1 axis drives osteoclastogenesis during bone remodelling.
- Pharmacological targeting of ZBP1 with JNJ‐10311795 bidirectionally modulates bone remodelling.

## Graphical abstract

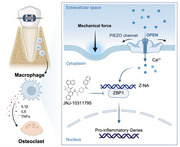

## 1 INTRODUCTION

Malocclusion is a highly prevalent oral condition worldwide, affecting not only masticatory function and increasing the risk of dental caries and periodontal disease, but also significantly impairing patients’ quality of life and psychosocial well‐being. Therefore, orthodontic treatment has become a cornerstone of modern oral healthcare.^1^, ^2^, ^3^, ^4^ The biological basis of such treatment is orthodontic tooth movement (OTM): the application of precise mechanical force to induce controllable remodelling in the periodontal tissues, thereby restoring dental function and aesthetics.^5^, ^6^ However, a long‐standing clinical bottleneck is the significant inter‐individual variability and unpredictability in the rate of OTM.^7^ This variability complicates the treatment plan, prolongs the course of treatment, and increases the risk of root resorption and alveolar bone loss.^7^, ^8^ In fact, how to achieve spatiotemporal precision in controlling bone remodelling represents not only a challenge in orthodontics but also a central and shared problem in orthopaedic regenerative fields, including fracture healing and distraction osteogenesis.

In recent years, the understanding of OTM mechanisms has evolved from a simple biomechanical model to a complex ‘osteoimmunology’ paradigm, wherein sterile inflammation triggered by mechanical stress orchestrates the balance between osteoclasts and osteoblasts.^5^, ^9^, ^10^, ^11^ Within this paradigm, macrophages have emerged as pivotal mechanosensory and immunomodulatory hubs.^12^ They not only sense mechanical microenvironmental changes, but also amplify inflammatory signals through polarization and the release of cytokine such as TNF‐α, IL‐1β and receptor activator of nuclear factor kappa‐B ligand (RANKL). Furthermore, they directly differentiate into osteoclasts or recruit and activate osteoclast precursors.^5^, ^9^, ^13^ The recognition of Piezo1, a mechanosensitive ion channel, as a key force sensor in immune cells represents a major advance. It provides a molecular basis for mechano‐immunology, the study of how mechanical forces direct immune cell function.^13^ Piezo1‐mediated Ca^2^^+^ influx in macrophages has been shown to regulate diverse processes from cytokine storm to tissue repair, highlighting its context‐dependent role as a central converter of physical signals into biological responses in the skeletal system.^14^, ^15^ This channel is functionally expressed in macrophages, and its activation can affect cell proliferation, polarization and cytokine production.^16^, ^17^, ^18^, ^19^ It is worth noting that the role of Piezo1 is context‐dependent:^20^ in the tissue regeneration environment, it can promote the formation of a pro‐repair macrophage phenotype;^21^, ^22^ but under excessive mechanical stress conditions (e.g., excessive orthodontic force), the activation of Piezo1 drives harmful pro‐inflammatory M1 polarization.^17^, ^23^ Macrophage Piezo1 is likely to be a key mechanosensor in OTM, but the downstream pathways coupling its activation still need to be further explored.

While Piezo1 is established as a proximal mechanosensor, the critical intracellular signalling pathways that specifically link its activation to the osteoclastogenic program in sterile conditions remain unknown. This study addressed this gap by identifying ZBP1 as the crucial downstream effector. Traditionally, ZBP1 (Z‐DNA binding protein 1) is defined as a cytoplasmic nucleic acid sensor that detects viral Z‐RNA and activates potent innate immune and cell death pathways.^24^, ^25^, ^26^, ^27^, ^28^ Here, we repositioned ZBP1 beyond its canonical role in host defence, revealing its function as a novel sterile mechanical stress sensor in macrophages. We demonstrated that mechanical activation of Piezo1 triggers upregulation of ZBP1, which in turn nucleates a signalling complex essential for driving pro‐inflammatory cytokine production and osteoclast differentiation. This Piezo1–ZBP1 axis thus established a previously unrecognized mechano‐immune pathway that directly couples orthodontic force to bone resorption.

Furthermore, we translated this mechanistic insight into a therapeutic strategy. Through virtual screening, we identified JNJ‐10311795 as a potent modulator of ZBP1 function. Pharmacological intervention with this compound effectively decoupled mechanical force from detrimental bone loss, allowing precise modulation of OTM rates. Notably, the same compound could be used to accelerate fracture healing by favourably shifting the bone remodelling balance toward formation. Therefore, our work not only reveals ZBP1 as a fundamental component of sterile mechanotransduction but also establishes the Piezo1–ZBP1 axis as a versatile therapeutic target for achieving predictable bone remodelling across a spectrum of mechanically driven clinical contexts, from orthodontics to orthopaedic repair.

## 2 MATERIALS AND METHODS

### 2.1 Human PDL sample collection

**FIGURE 1:**
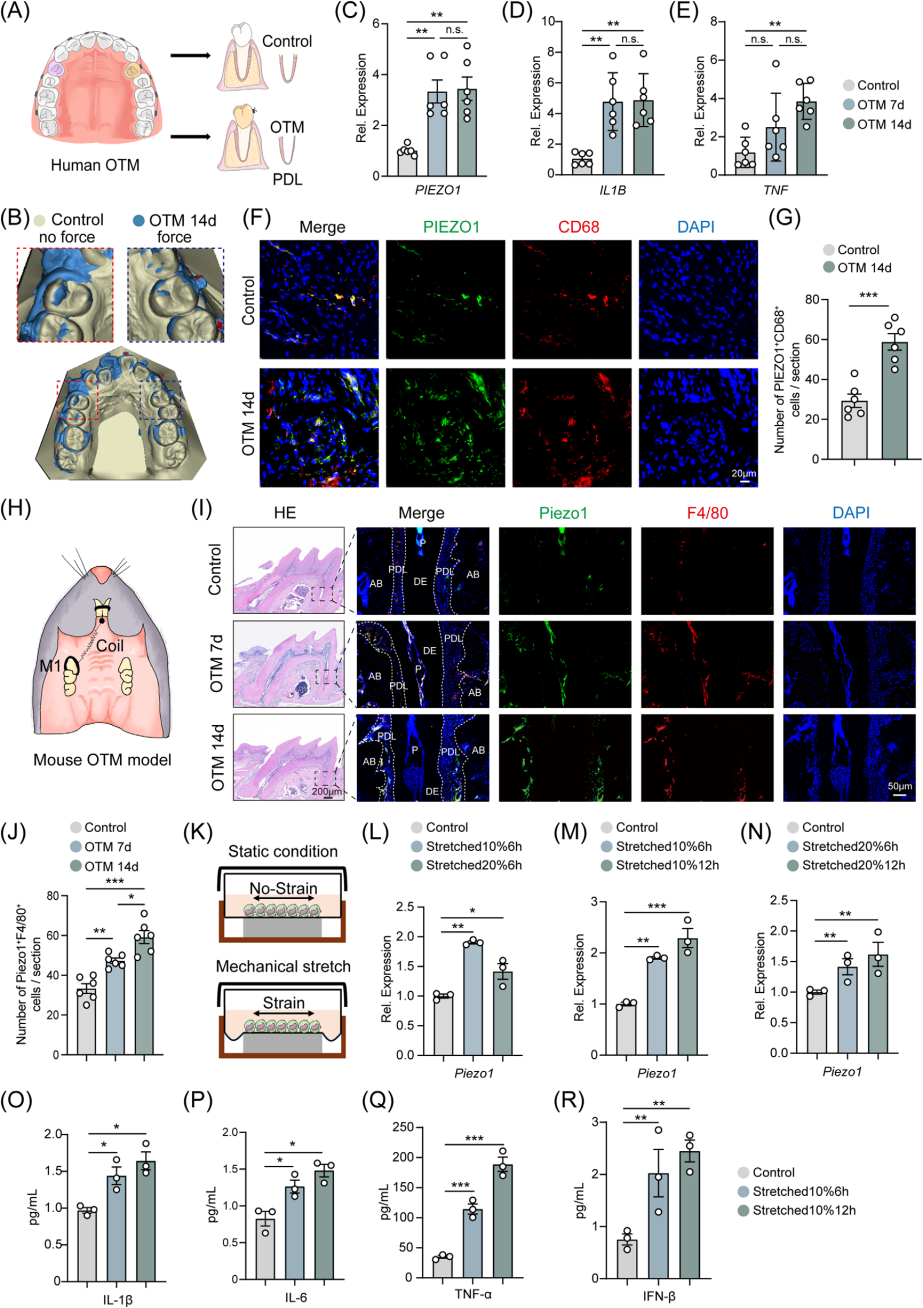
Mechanical force activates Piezo1 in macrophages and modulates the inflammatory responses during OTM. (A) Schematic diagram of the human orthodontic force application. (B) Superimposition of intraoral scan models before and after orthodontic force application. (C–E) qPCR analysis of *PIEZO1*, *IL1B* and *TNF* mRNA expression in human PDL tissues from BLANK (non‐loaded), 7‐day and 14‐day force‐loaded groups (*n *= 6). (F) Representative immunofluorescence images showing infiltration of PIEZO1^+^CD68^+^ cells in the PDL of human after 14 days of force application. (G) Quantification of PIEZO1^+^CD68^+^ cell numbers per section (*n *= 6). (H) Schematic of the mouse orthodontic tooth movement (OTM) model. (I) Representative hematoxylin and eosin (H&E) stained sections and immunofluorescence images showing infiltration of Piezo1^+^F4/80^+^ cells in the periodontium of mice after 7 and 14 days of force application. AB, alveolar bone; PDL, periodontal ligament; DE, dentin; P, tooth pulp. Scale bar of H&E staining images: 200 µm; Scale bar of immunofluorescence staining images: 50 µm. (J) Quantification of Piezo1^+^F4/80^+^ cell numbers per section at 0, 7, and 14 days (*n *= 6). (K) Schematic of the application of tensile stress to cells. (L–N) qPCR analysis of *Piezo1* mRNA expression in bone marrow‐derived macrophages (BMDMs) subjected to cyclic tensile strain (10% or 20% elongation) for 6 or 12 h (*n *= 3). (O‐R) ELISA measurement of IL‐1β, IL‐6, TNF‐α and IFN‐β levels in BMDMs under the same mechanical loading conditions (*n *= 3). All data are presented as mean ± SD (n.s., no significance, *p < 0.05, **p < 0.01, ***p < 0.001).

This study obtained human PDL samples with a self‐controlled, split‐mouth design. On one side, the first premolar was not subjected to orthodontic force, serving as the non‐loaded control. The contralateral first premolar was bonded using Damon Q self‐ligating brackets (ORMCO) and received a continuous force by engaging a 0.014‐inch nickel‐titanium archwire. The premolars were extracted at 7 or 14 days after force application (Figure 1A). PDL tissues were carefully harvested from the root surfaces of the extracted teeth using a sterile scalpel, immediately snap‐frozen in liquid nitrogen, and stored at ‐80°C until further use.

This study was approved by the Ethics Committee of Nanjing Medical University (PJ2021‐058‐01). All patient data were anonymized for privacy protection. Inclusion criteria: (1) age 12–25 years; (2) moderate to severe dental crowding requiring extraction of the maxillary first premolars for orthodontic treatment; (3) healthy periodontal tissues with a probing depth ≤ 3 mm and no history of periodontal disease; (4) absence of systemic diseases or long‐term medication use (e.g., anti‐inflammatory drugs, bisphosphonates) that could influence bone metabolism. Exclusion criteria included: (1) root resorption, tooth defects, or caries; (2) history of orthodontic treatment or maxillofacial surgery; (3) pregnancy or lactation.

### 2.2 Animals and Ethical Approval

This study used male C57BL/6J wild‐type (WT), *Piezo1* ^f/f^;*Cx3cr1‐*cre (hereafter *Piezo1* ^cko^) (Figure S1), and *Zbp1* ^f/f^;*Cx3cr1‐*cre (hereafter *Zbp1* ^cko^) (Figure S2) mice. All mice aged 8–10 weeks were obtained from GemPharmatech Co. Ltd. A soft diet was provided after OTM induction.

Genotyping was performed by PCR analysis. The primers, reaction mixture, and thermal cycling conditions are detailed in Tables S1–3. The expected fragment sizes for distinguishing wild‐type and floxed alleles are provided in Table S1. To confirm efficient deletion of the target genes in macrophages, bone marrow‐derived macrophages (BMDMs) isolated from *Piezo1* ^f/f^, *Piezo1* ^cko^, *Zbp1* ^f/f^ and *Zbp1* ^cko^ mice were analysed at mRNA and protein levels.

All procedures were approved by the Animal Ethics Committee of Nanjing Medical University (IACUC‐2309028 and IACUC‐2503016).

### 2.3 Mouse Model of OTM

Mice were anaesthetized via intraperitoneal injection of sodium pentobarbital (50 mg/kg body weight) prior to all treatment procedures. A nickel‐titanium coil spring (5 mm in length; IMD) was ligated between the maxillary first molar (M1) and incisor using 0.020‐inch stainless steel wire to deliver an initial, continuous orthodontic force of 30 g (Figure 1H).^29^, ^30^ The wire was carefully secured around the cervical region of each tooth to prevent soft tissue injury. Loading durations were 3, 7 and 14 days. The contralateral side served as the internal control. The integrity and correct position of the orthodontic appliance were checked daily. Postoperatively, mice were administered analgesics and monitored daily for health and weight. A soft diet was provided throughout the experimental period to ensure adequate nutrition.

### 2.4 Mouse Femoral Fracture Healing Model

Mice were anaesthetized as described before. A medial parapatellar approach was used under sterile conditions to expose the femoral intercondylar notch. After drilling a pilot hole, a 0.6‐mm Kirschner wire (K‐wire) was inserted into the medullary cavity. Subsequently, a flap was incised to expose the midshaft of the femur, and an ultrasonic bone scalpel was used to create a standardized fracture. The K‐wire was adjusted to securely span the fracture site, providing stable intramedullary fixation, and the joint capsule and skin were sutured.^31^ Mice were allowed free ambulation postoperatively.

### 2.5 Cell Culture and Stimulation

BMDMs were harvested from mouse femurs and differentiated in complete medium (α‐MEM, Gibco) supplemented with 10% FBS (ScienCell), 1% penicillin/streptomycin (Gibco), and 25 ng/mL M‐CSF (PeproTech) for 5 days. For mechanical stimulation experiments, differentiated BMDMs were seeded onto BioFlex 6‐well plates (Flexcell) that had been precoated with type I collagen (Sigma‐Aldrich). After 24 hours of adhesion, BMDMs were subjected to cyclic uniaxial tensile strain (10% or 20% elongation, 0.5 Hz) for 6 or 12 h using a Flexcell‐5000T system (Flexcell), with control cells cultured on identical plates but without mechanical stretch.^32^ For all other non‐mechanical experiments, BMDMs were seeded onto standard 6‐well tissue culture plates (Corning) and maintained under static conditions with the respective compounds or vehicle control. For calcium‐free conditions, BMDMs were first cultured in normal complete medium. Before mechanical stretching, cells were washed with Ca^2^^+^‐free PBS and then transferred to Ca^2^^+^‐free DMEM (Gibco, cat# 21068028) supplemented with 10% dialyzed FBS (Gibco, cat# 26400044), 1% penicillin‐streptomycin, and 2 mM EGTA (Sigma‐Aldrich, cat# E3889) to chelate residual Ca^2^^+^. Cells were pre‐incubated in Ca^2^^+^‐free medium for 30 min before force application, and the medium was maintained during the entire stretching period.

### 2.6 ELISA

The concentrations of IL‐1β, IL‐6, TNF‐α and IFN‐β in cell culture supernatants were analysed with commercial sandwich ELISA kits. Briefly, cell supernatants were collected and centrifuged to remove cellular debris. Following the manufacturer's protocol, 96‐well plates were coated with capture antibodies overnight at 4°C. After blocking with 1% BSA, appropriately diluted samples and recombinant cytokine standards, which were serially diluted in the complete culture medium from unstimulated cells to match the sample matrix, were added in duplicate and incubated. Subsequently, biotinylated detection antibodies, streptavidin‐horseradish peroxidase (HRP), and tetramethylbenzidine (TMB) substrate were sequentially added with thorough washing between steps. The reaction was terminated with sulphuric acid, and the absorbance was measured at 450 nm. Cytokine concentrations were determined by interpolating the absorbance values based on the standard curve.

### 2.7 Drug Screening

Structure‐based virtual screening was performed to identify small molecule inhibitors targeting the Zα domain of ZBP1. The crystal structure of the ZBP1 Zα domain was obtained from the RCSB Protein Data Bank (PDB ID: 3EYI). To account for conformational flexibility, AlphaFold2‐predicted models of the Zα domain were also used. Protein structure was preprocessed using PyMOL, including eliminating water molecules, adding hydrogen atoms, and repair of missing side chains. The nucleic acid‐binding pocket of the Zα domain was defined as the docking site. A library of 2,513 FDA‐approved drugs was retrieved from the ZINC database. All compounds were preprocessed using OpenBabel to generate PDBQT format files for docking. Semi‐flexible molecular docking was performed using AutoDock Vina. The docking grid box was centred at the Zα domain binding pocket. The binding free energy (ΔG, kcal/mol) was used as the primary scoring metric, with lower ΔG indicating more stable predicted binding. Compounds were ranked based on their ΔG values (Table S4). The top‐ranking compounds were visually inspected for favourable binding poses within the Zα domain pocket using PyMOL. The final candidate compounds were selected based on their docking scores, structural diversity, drug‐like properties and commercial availability for subsequent experimental validation.^33^

### 2.8 Pharmacological Treatments

Yoda1 (#5586, Tocris), JNJ‐10311795 (#HY‐122161, MCE), Cromoglicic acid (#HY‐B1619, MCE) and LY‐2584702 (#HY‐12493A, MCE) were dissolved in DMSO (10 mM for Yoda1; 1 mM for the others). Working concentrations were freshly diluted in complete cell culture medium right prior to cell treatment.

For in vitro studies, BMDMs were treated with Yoda1 (5 or 10 µM) as a Piezo1 agonist. For in vitro validation of the virtual screening hits, BMDMs were treated with JNJ‐10311795, Cromoglicic acid, or LY‐2584702 at concentrations ranging from 1 to 20 nM. For lentivirus‐mediated gene overexpression in vitro, BMDMs were transduced with Lv‐*Zbp1* or Lv‐NC at a multiplicity of infection (MOI) of 100 for 48 h before mechanical stretch application.

For in vivo studies, JNJ‐10311795 was diluted in a vehicle of 5% DMSO in corn oil to a working concentration (0.1 mg/mL). A dose of 1 mg/kg was administered via intraperitoneal injection on Days 7, 9 and 11 after OTM induction. For lentivirus‐mediated gene overexpression in vivo, *Zbp1*‐overexpressing lentivirus (Lv‐*Zbp1*) and control lentivirus (Lv‐NC) were purchased from GentleGen Technologies Co. (Suzhou, China). Viruses were diluted in sterile PBS to a titre of 1 × 10^8^ transduction units (TU)/mL. A dose of 50 µL (5 × 10^6^ TU) was administered via tail vein injection on Days 7, 9 and 11 following OTM induction.

### 2.9 Micro‐CT Analysis

Following fixation in 4% paraformaldehyde, maxillae and femurs were scanned using a vivaCT80 system (Scanco Medical) at 55 kV, 72 µA, and an isotropic resolution of 15.6 µm. For OTM distance measurement, three‐dimensional reconstruction was performed using Mimics 20.0 software (Materialise). The distance between the distobuccal prominence of the left maxillary first molar and the mesiobuccal prominence of the second molar was measured to quantify OTM distance (Figure S3A–C). Each sample was measured three times by the same operator, and the average value was used to minimize measurement error.^34^ For bone microstructure analysis, the region of interest (ROI) was defined as the mid‐distal region of the first molar distobuccal root, encompassing 20 consecutive sections (Figure S3D). Using CT Analyzer software (Bruker), a consistent global threshold was applied to all samples to segment mineralized tissue from bone marrow and soft tissue. Measured trabecular indices, including bone volume fraction (BV/TV), trabecular thickness (Tb.Th), trabecular number (Tb.N), and trabecular separation (Tb.Sp) were calculated.^35^ For the fracture model, the ROI was defined as the entire callus area. Callus volume was measured using the same method.

### 2.10 Histological Analysis

Maxillae were fixed for 48 h, decalcified for 3 weeks (media changed every 3 days), and embedded. Paraffin sections (4 µm) were cut along the mesiodistal plane of the left maxillary first molar, while frozen sections (8 µm) were prepared after cryoprotection in 30% sucrose and OCT embedding. Paraffin sections underwent hematoxylin and eosin (H&E), Tartrate‐Resistant Acid Phosphatase (TRAP) or safranin O/fast green (SO/FG) staining. For immunofluorescence, frozen sections were blocked with 10% BSA and incubated overnight at 4°C with primary antibodies against CD68 (1:50, #ab955, Abcam), F4/80 (1:100, #ab16911, Abcam), Piezo1 (1:100, #28511‐1‐AP, ProteinTech), iNOS (1:100, #ab15323, Abcam), ZBP1 (1:400, #AG‐20B‐0010, AdipoGen), and Z‐DNA(1:400, #Ab00783‐3.0, Absolute Antibody), followed by appropriate fluorochrome‐conjugated secondary antibodies and DAPI counterstaining. The anti‐Z‐DNA antibody recognizes left‐handed Z‐conformation nucleic acids, including both Z‐DNA and Z‐RNA, with high specificity. All images were acquired on a Leica DM6B microscope.

### 2.11 RNA extraction and qPCR

Total RNA was extracted from tissues and BMDMs using TRIzol reagent. cDNA was synthesized using a reverse transcription kit (Vazyme). qPCR was performed using SYBR Green chemistry with primers listed in Table S5, following the reaction conditions detailed in Tables S6 and S7. Gene expression was normalized to *Gapdh* and calculated using the 2^ΔΔCt^ method.

### 2.12 Cellular thermal shift assay (CETSA)

CETSA was performed to assess whether JNJ‐10311795 directly binds to ZBP1 in intact cells.^36^, ^37^ BMDMs were seeded onto BioFlex plates and cultured. They were treated with 10 nM JNJ‐10311795 or DMSO (0.1% v/v) for 1 h, followed by mechanical stretching using the Flexcell‐5000T system (10% elongation, 0.5 Hz, 6 h). After stretching, BMDMs were harvested, resuspended in PBS (2× 10^7^ cells/mL), and heated for 3 min across a 35°C–70°C gradient (35°C, 40°C, 45°C, 50°C, 55°C, 60°C, 65°C, 70°C) in a thermal cycler. After heating, BMDMs were lysed via two freeze‐thaw cycles. Lysates were centrifuged and the supernatants containing soluble proteins were collected. Western blot was performed as described in Section 2.13 using an anti‐ZBP1 antibody. Band intensities were quantified using ImageJ. The soluble ZBP1 fraction at each temperature was expressed as a percentage of the 35°C signal. Thermal denaturation curves were plotted as the percentage of soluble protein against temperature to illustrate the thermal stability shift of ZBP1 upon JNJ‐10311795 treatment.

### 2.13 Western blot

Proteins were isolated from BMDMs with RIPA lysis buffer supplemented with PMSF. Protein concentrations were measured using a BCA kit. Equal protein loads (20–30 µg) were resolved on 10% SDS‐PAGE, electrotransferred onto PVDF membranes, and then blocked in 5% non‐fat milk. Membranes were incubated overnight with primary antibodies against ZBP1 (1:1000, #AG‐20B‐0010, AdipoGen) and GAPDH (1:5000, #10494‐1‐AP, ProteinTech), followed by HRP‐conjugated secondary antibodies incubation. Immunoreactive bands were detected using ECL reagent, with GAPDH serving as a loading control.

### 2.14 RNA Sequencing

RNA from mechanically loaded BMDMs (*Piezo1* ^f/f^ vs. *Piezo1* ^cko^) was sequenced on an Illumina platform. After adapter trimming and quality filtering with fastp, the remaining high‐quality reads were mapped to the GRCm38.p4 genome using HISAT2. Gene‐level counts were fed into edgeR for differential expression analysis, with significance defined as |Fold Change|≥ 2 and FDR ≤ 0.05. Enrichment analysis of GO terms and KEGG pathways was then performed on genes meeting these criteria (FDR ≤ 0.05). The raw sequence data are available in the Genome Sequence Archive under the accession code PRJCA057077 (https://ngdc.cncb.ac.cn/bioproject/browse/PRJCA057077).

### 2.15 Calcium Imaging

Intracellular Ca^2^^+^ flux was measured by flow cytometry using the Ca^2^^+^‐sensitive probe Fluo‐4 AM. BMDMs from *Piezo1* ^f/f^ and *Piezo1* ^cko^ mice were resuspended in HBSS at 1 × 10^6^ cells/mL and loaded with 5 µM Fluo‐4 AM (Thermo Fisher Scientific, Cat. No. F14201) containing 0.02% Pluronic F‐127. BMDMs were then washed with HBSS and resuspended in fresh HBSS. Fluorescence was detected on a flow cytometer (BD LSRFortessa, USA) with excitation at 488 nm and emission collected using a 530/30 nm bandpass filter. A minimum of 10 000 events per sample were collected, and data were gated on live cells based on forward and side scatter. Baseline fluorescence was recorded for 30 s, followed by addition of Yoda1 (10 µM) or vehicle control. Fluorescence intensity was continuously recorded at 1‐s intervals for at least 480 s. Data were analysed using FlowJo software. Calcium flux was calculated asΔF/F_0_ = (F—F_0_)/F_0_, where F_0_ is the mean baseline fluorescence intensity.

### 2.16 Osteoclast Differentiation Assay

BMDMs were cultured in complete medium containing 25 ng/mL RANKL (#315‐11, PeproTech) for 5 days, with medium refreshed every 3 days. Osteoclasts were identified by TRAP staining and counted (multinucleated cells with≥3 nuclei).

### 2.17 Statistics

Experimental data are presented as the mean ± standard deviation (SD). The sample size (*n*) is specified in the figure legends, which stands for the biological replicates of independent experimental procedures. Statistical analyses were carried out with GraphPad Prism software (version 9.0). The Shapiro–Wilk test was used to test for normality, while the Brown–Forsythe test was used to assess the homogeneity of variances. For the comparison between two groups, an unpaired, two‐tailed Student's *t*‐test was adopted when variances were equal. In instances where the variances were unequal, Welch's corrected *t*‐test was applied instead. When comparisons involved three or more groups, one‐way analysis of variance (ANOVA) was performed. Correlation analysis was performed by Spearman's rank correlation coefficient. A *p*‐value less than 0.05 was considered statistically significant. Significance levels are indicated in the figures as *p < 0.05, **p < 0.01, ***p < 0.001; n.s. indicates not significant.

## 3 RESULTS

### 3.1 Mechanical force activates Piezo1 in macrophages and modulates the inflammatory responses during OTM

To investigate mechanosensation during OTM, we collected human periodontal ligament (PDL) samples from orthodontic patients to establish an experimental model (Figure 1A). Baseline characteristics are shown in Table S8. Superimposition of intraoral scan models before and after orthodontic force application confirmed significant tooth displacement on the force‑applied side (Figure 1B). PDL tissues from the force‑applied side (OTM) and the control side (Control) were harvested for quantitative polymerase chain reaction (qPCR) and immunofluorescence analysis. Results showed that *PIEZO1*, *IL1B* and *TNF* mRNA expression was significantly upregulated in mechanically stressed PDL at 7 and 14 days after force application (Figure 1C–E). In addition, the number of Piezo1^+^ macrophages in the PDL on the force‐applied side markedly increased at day 14 of OTM (Figure 1F,G). To further explore the clinical relevance of Piezo1 in force‐induced inflammation, we performed correlation analyses between *PIEZO1* and pro‐inflammatory cytokine expression in human PDL samples, which revealed that *PIEZO1* levels were significantly positively correlated with both *IL1B* and *TNF* mRNA levels (Figure S4A,B). These findings extend previous reports of *Piezo1* expression in murine PDL,^16^, ^38^, ^39^ indicating that orthodontic force activates PIEZO1‐associated inflammatory signalling in human PDL. In a mouse OTM model (Figure 1H), 7 and 14 days of force application induced characteristic periodontal remodelling, including widened PDL space and disorganized collagen fibres (Figure 1I). Immunofluorescence further showed substantial infiltration of F4/80^+^ macrophages and Piezo1^+^ macrophages in mechanically stimulated periodontium. Quantification further revealed that Piezo1^+^F4/80^+^ macrophages increased progressively from Day 0 to Day 7 and remained significantly elevated at Day 14 after force application (Figure 1I,J).

Corresponding in vitro studies demonstrated that cyclic tensile strain directly upregulated *Piezo1* expression in BMDMs, with maximal induction at 10% elongation for 12 h (Figure 1K–N). Mechanical loading at this optimal condition also significantly enhanced expression of IL‐1β, IL‐6, TNF‐α and IFN‐β, showing greater induction at 12 versus 6 h (Figure 1O–R). These findings establish that mechanical force enhances *Piezo1* expression and promotes macrophage‐mediated inflammatory responses during OTM.

### 3.2 Macrophage‐specific deletion of *Piezo1* decelerates OTM and alters bone remodelling

**FIGURE 2:**
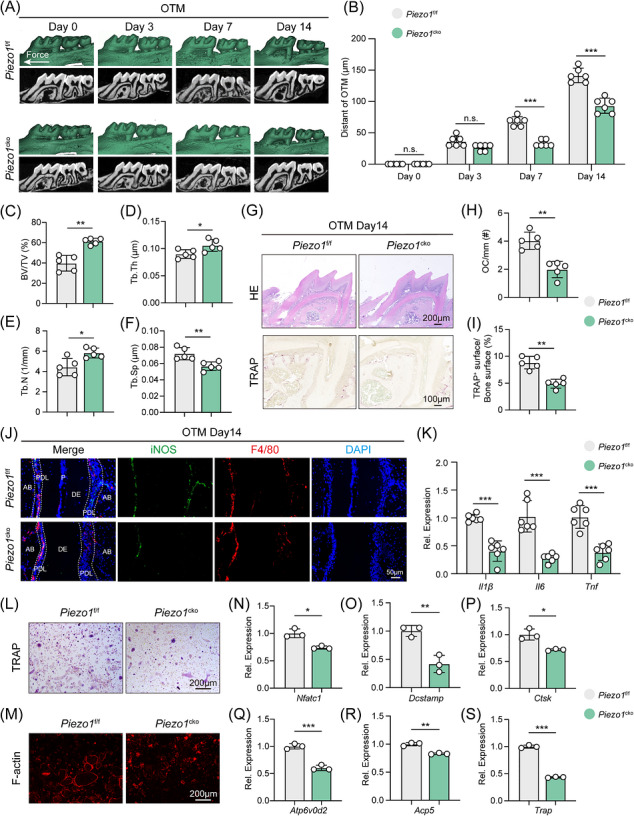
Macrophage‐specific *Piezo1* deletion impairs OTM and attenuates the inflammatory response. (A) Representative three‐dimensional micro‐CT reconstructions of the maxillae from wild‐type (*Piezo1* ^f/f^) and macrophage‐specific *Piezo1* knockout (*Piezo1* ^cko^) mice after 0, 3, 7, and 14 days of OTM. (B) Quantification of OTM distance in *Piezo1* ^f/f^ and *Piezo1* ^cko^ mice at the indicated time points (*n *= 6). (C–F) Micro‐CT analysis of alveolar bone microarchitecture on the tension side, including trabecular thickness (Tb.Th), bone volume/total volume (BV/TV), trabecular number (Tb.N), and trabecular separation (Tb.Sp) (*n *= 5). (G) Representative H&E and TRAP‐stained sections of the periodontium in *Piezo1* ^f/f^ and *Piezo1* ^cko^ mice after 14 days of OTM. Scale bar of H&E staining images: 200 µm; Scale bar of TRAP staining images: 100 µm. (H, I) Quantification of osteoclast numbers per bone perimeter and TRAP‐positive surface relative to the bone surface (*n *= 5). (J) Representative immunofluorescence images and quantification of F4/80^+^iNOS^+^ cell infiltration in the periodontium of *Piezo1* ^f/f^ and *Piezo1* ^cko^ mice. Scale bar: 50 µm. (K) qPCR analysis of *Il1β*, *Il6* and *Tnf* mRNA expression in periodontal tissues from *Piezo1* ^f/f^ and *Piezo1* ^cko^ mice after 14 days of OTM (*n *= 6). (L) TRAP staining of osteoclasts cultured from *Piezo1* ^f/f^ and *Piezo1* ^cko^ mice. Scale bar: 200 µm. (M) Rhodamine‐conjugated phalloidin staining of F‐actin belt in osteoclasts cultured from *Piezo1* ^f/f^ and *Piezo1* ^cko^ mice. Scale bar: 200 µm. (N‐S) qPCR analysis of *Nfatc1*, *Dcstamp, Ctsk, Atp6v0d2, Acp5* and *Trap* mRNA expression in osteoclasts cultured from *Piezo1* ^f/f^ and *Piezo1* ^cko^ mice after 14 days of OTM (*n *= 3). All data are presented as mean ± SD (n.s., no significance, *p < 0.05, **p < 0.01, ***p < 0.001).

To investigate the crucial role of macrophage Piezo1 in force‐induced bone remodelling, we conducted studies using a macrophage‐specific *Piezo1* knockout mouse model (*Piezo1* ^cko^) (Figure S1). Analysis of OTM revealed that although tooth displacement progressively increased in all mice over time, *Piezo1* ^cko^ mice exhibited significantly reduced movement distance compared to *Piezo1* ^f/f^ controls at Days 7 and 14 (Figure 2A,B). This finding provides genetic evidence for the first time that the macrophage Piezo1 is a key molecular switch that mediates the continuous movement of teeth in response to orthodontic force, and its normal function is essential for achieving the expected orthodontic treatment effect.

Further micro‐CT analysis uncovered the microstructural basis of this phenomenon. On the 14th day of treatment, Tb.Th, BV/TV and Tb.N of the alveolar bone distal to first molar in *Piezo1* ^cko^ mice were significantly increased, while Tb.Sp was reduced (Figure 2C–F). These data show that Piezo1 actively facilitates the orthodontic treatment process by precisely regulating the dynamic balance between alveolar bone resorption and formation, thus creating the necessary space for tooth movement through bone remodelling.

Histological examination on the 14th day showed that compared with *Piezo1* ^f/f^ mice, the periodontal tissue remodelling process of *Piezo1* ^cko^ mice was weakened, evidenced by reduced PDL space widening and fewer bone resorption lacunae (Figure 2G). TRAP staining confirmed a significant decrease in osteoclast numbers along the alveolar bone in *Piezo1* ^cko^ mice (Figure 2G–I). While infiltration of F4/80^+^iNOS^+^ macrophages in *Piezo1* ^cko^ periodontal tissues was reduced (Figure 2J), the expression of pro‐inflammatory cytokines (*Il1β*, *Il6*, *Tnf*) was also markedly reduced (Figure 2K), demonstrating that macrophage Piezo1 is essential for inflammatory activation during OTM. Furthermore, when BMDMs from *Piezo1* ^f/f^ and *Piezo1* ^cko^ mice were induced to undergo osteoclastogenesis, *Piezo1* deletion attenuated osteoclast formation (Figure 2L,M) and downregulated osteoclast‐related gene expression (Figure 2N–S).

Combined micro‐CT and histological analyses demonstrated enhanced callus formation and accelerated fracture healing in *Piezo1* ^cko^ mice at Day 14. In contrast, *Piezo1* ^f/f^ calluses exhibited more cartilage with active osteoclasts at the remodelling front, indicative of an intermediate stage of endochondral ossification (Figure S5). This suggests that macrophage‐specific *Piezo1* deletion accelerates healing, likely due to reduced osteoclastogenesis which would otherwise prolong the cartilage resorption phase.

### 3.3 Piezo1 activates the ZBP1 signalling pathway in macrophages

**FIGURE 3:**
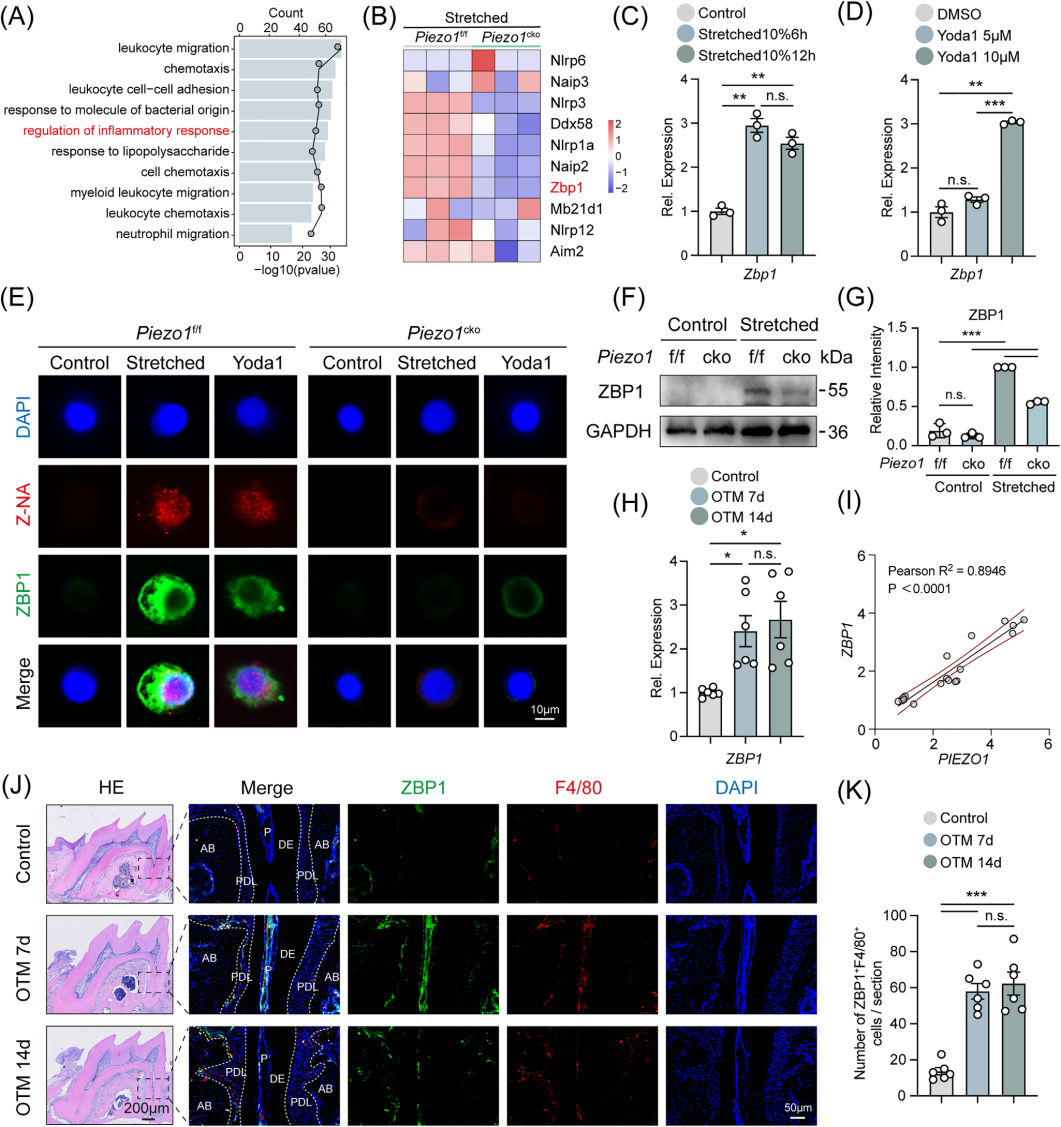
Piezo1 activates the ZBP1 signalling pathway in macrophages and is correlated with human OTM. (A) Gene Ontology (GO) enrichment analysis of down‐regulated differentially expressed genes in BMDMs from force‐treated *Piezo1* ^f/f^ versus *Piezo1* ^cko^ mice. (B) Heatmap showing transcriptomic changes of inflammatory response related genes in *Piezo1* ^cko^ BMDMs compared to *Piezo1* ^f/f^ controls upon mechanical stimulation. (C) qPCR analysis of *Zbp1* mRNA expression in BMDMs subjected to cyclic tensile strain (10%) for 6 or 12 hours (*n *= 3). (D) qPCR analysis of *Zbp1* mRNA expression in BMDMs treated with increasing concentrations of Yoda1 (0, 5, 10 µM) for 24 hours (*n *= 3). (E) Representative immunofluorescence images of ZBP1 protein (green) and Z‐nucleic acids (red) in *Piezo1* ^f/f^ and *Piezo1* ^cko^ BMDMs treated with mechanical stretch (10%, 6 h) or the Piezo1 agonist Yoda1 (10 µM). Nuclei are stained with DAPI (blue). Scale bar: 10 µm. (F) Western blot analysis of ZBP1 protein expression in *Piezo1* ^f/f^ and *Piezo1* ^cko^ BMDMs with or without mechanical stretch. (G) Densitometric quantification for ZBP1 protein expression. (H) qPCR analysis of *ZBP1* expression in human periodontal ligament tissues. (I) Correlation plot showing the positive correlation between *PIEZO1* and *ZBP1* expression levels in human samples. (J) Representative immunofluorescence images showing infiltration of ZBP1^+^F4/80^+^ cells in the periodontium of mice after 7 and 14 days of OTM. (K) Quantification of ZBP1^+^F4/80^+^ macrophage staining in mouse OTM model (time course 0, 7, 14d). All data are presented as mean ± SD (*p < 0.05, **p < 0.01, ***p < 0.001).

To elucidate the downstream mechanisms by which macrophages transduce mechanical signals through Piezo1, we integrated transcriptomic profiling with in vitro functional assays. RNA sequencing of BMDMs from *Piezo1* ^f/f^ and *Piezo1* ^cko^ mice after mechanical stimulation showed that pathways related to leukocyte migration and inflammatory regulation were significantly enriched in *Piezo1* ^f/f^ cells (Figure 3A). Notably, the expression of innate immune sensors *Zbp1*, *Nlrp3* and *Naip2* was markedly down‐regulated in BMDMs of *Piezo1* ^cko^ mice (Figure 3B), suggesting ZBP1 may function as a key downstream effector molecule for Piezo1‐mediated mechanotransduction.

We next validated the mechanical regulation of *Zbp1* in vitro. Cyclic tensile strain significantly upregulated the mRNA level of *Zbp1*, with peak induction at 6 h (Figure 3C). In addition, Piezo1‐specific agonist Yoda1^40^ stimulation enhanced the mRNA expression of *Zbp1* in *Piezo1* ^f/f^ BMDMs (Figure 3D). Immunofluorescence analysis showed that both mechanical stretch and Yoda1 strongly promoted the accumulation of ZBP1 protein and the formation of Z‐nucleic acids in *Piezo1* ^f/f^ BMDMs, whereas these fluorescence signals were substantially weakened in *Piezo1* ^cko^ cells (Figure 3E). Western blotting further confirmed that stretch‐induced ZBP1 upregulation is Piezo1‐dependent (Figure 3F, 3G).

To further validate the clinical relevance of the Piezo1‐ZBP1 axis, human PDL tissues were analysed. qPCR analysis showed that *ZBP1* mRNA levels were significantly upregulated in human PDL tissues after orthodontic force application (Figure 3H). Correlation analysis further revealed a strong positive correlation between *PIEZO1* and *ZBP1* expression levels in human samples (Figure 3I). We further performed correlation analyses and found that *ZBP1* levels were also significantly positively correlated with *TNF* mRNA levels in human PDL samples (Figure S4C,D). Consistently, immunofluorescence staining of mouse periodontal tissues showed a time‐dependent increase in ZBP1^+^F4/80^+^ macrophages during OTM, with significant elevation observed at both Day 7 and Day 14 (Figure 3J, K).

### 3.4 ZBP1 mediates Piezo1‐dependent osteoclastogenesis and OTM

**FIGURE 4:**
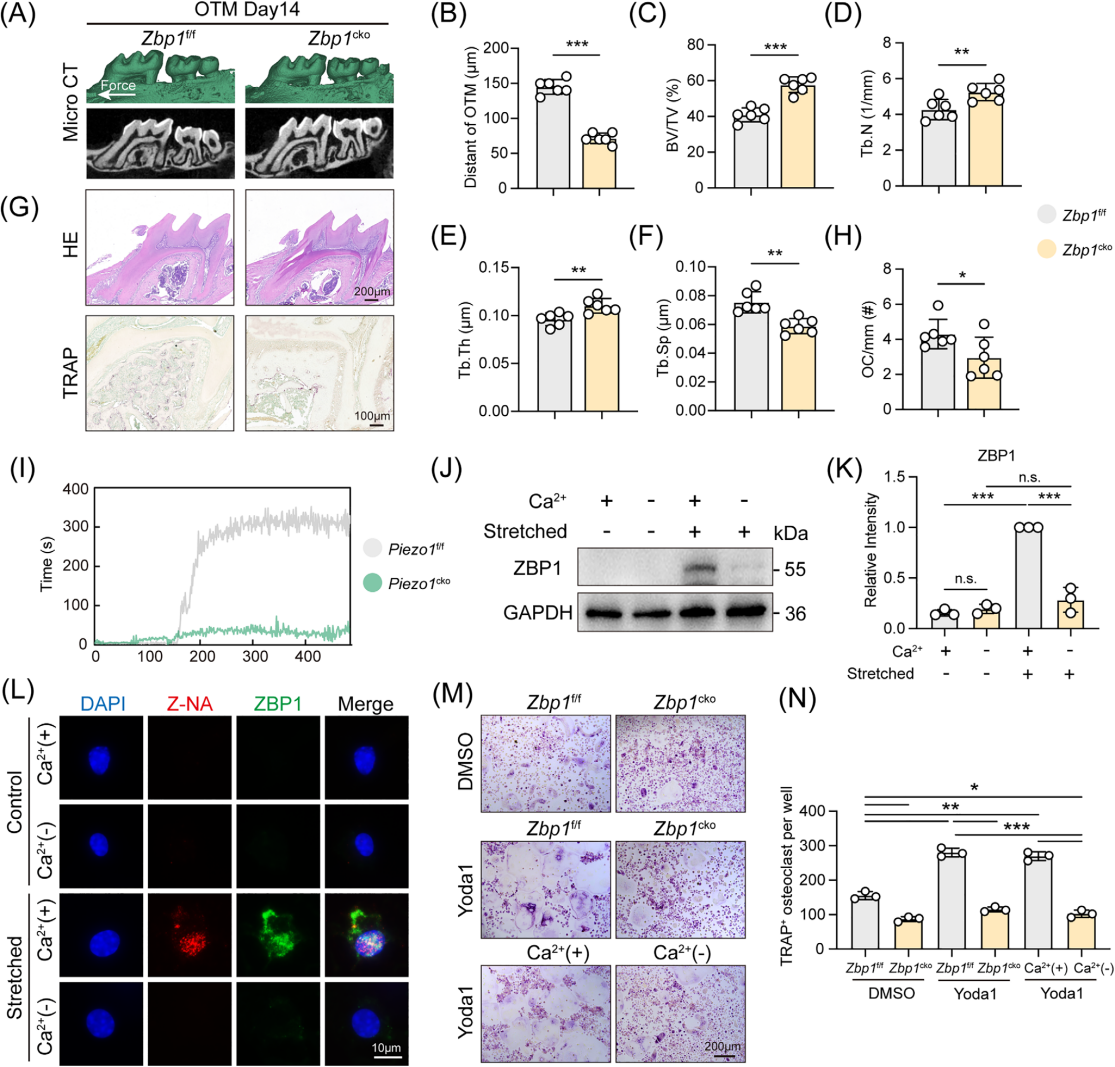
ZBP1 mediates Piezo1/Ca^2^^+^‐dependent osteoclastogenesis and orthodontic tooth movement. (A and B) Representative three‐dimensional micro‐CT reconstructions of the maxillae from *Zbp1* ^f/f^ and macrophage‐specific *Zbp1* knockout (*Zbp1* ^cko^) mice after 14 days of OTM and quantification of OTM distance (*n *= 6). (C–F) Micro‐CT analysis of alveolar bone microarchitecture on the tension side, including BV/TV, Tb.N, Tb.Th, and Tb.Sp (*n *= 6). (G and H) Representative H&E and TRAP‐stained sections of the periodontium in *Zbp1* ^f/f^ and *Zbp1* ^cko^ mice after 14 days of OTM and quantification of osteoclast numbers per bone perimeter. Scale bar of H&E staining images: 200 µm; Scale bar of TRAP staining images: 100 µm (*n *= 6). (I) Flow cytometry analysis of intracellular Ca^2^^+^ flux (measured by Fluo‐4 AM fluorescence) in *Piezo1* ^f/f^ and *Piezo1* ^cko^ BMDMs in response to Yoda1 (10 µM) stimulation. (J and K) Western blot analysis of ZBP1 protein expression in BMDMs under conditions of mechanical stretch and/or high extracellular Ca^2^^+^. (L) Representative immunofluorescence images showing colocalization of Z‐nucleic acids (red) and ZBP1 protein (green) in BMDMs under mechanical stretch, with or without extracellular Ca^2^^+^. Nuclei are stained with DAPI (blue). Scale bar: 10 µm. (M and N) Representative TRAP staining and quantification of TRAP^+^ osteoclast per well from *Zbp1* ^f/f^ and *Zbp1* ^cko^ BMDMs treated with Yoda1 and/or Ca^2^^+^ (*n *= 3). All data are presented as mean ± SD (*p < 0.05, **p < 0.01, ***p < 0.001).

To directly test the functional requirement of ZBP1 in OTM, we generated macrophage‐specific *Zbp1* knockout mice (*Zbp1* ^cko^) (Figure S2). The deletion of *Zbp1* significantly inhibited OTM. After 14 days of force application, micro‐CT analysis showed markedly reduced tooth displacement in *Zbp1* ^cko^ mice compared to *Zbp1* ^f/f^ controls (Figure 4A,B). Analysis of bone microarchitectural parameters on the tension side of the moved tooth showed that bone formation was preserved in *Zbp1* ^cko^ mice, evidenced by significant increases in BV/TV, Tb.N and Tb.Th, along with a decrease in Tb.Sp (Figure 4C–F). Consistent with this phenotype, histological examination showed that the tissue remodelling process of *Zbp1* ^cko^ mice was attenuated, which was characterized by a decrease in the number of osteoclasts along the bone surface (Figure 4G,H). These findings indicate that *Zbp1* deficiency disrupts the immune microenvironment essential for macrophage‐mediated bone resorption.

To find out the causal relationship between the mechanosensitive activation of Piezo1 and ZBP1 and subsequent osteoclastogenesis, we focused on the pivotal role of the Ca^2^^+^ signalling pathway. Ca^2^^+^ influx assays showed that Yoda1 induced a rapid increase in intracellular Ca^2^^+^ concentration in BMDMs from *Piezo1* ^f/f^ mice, an effect that was markedly blunted in *Piezo1* ^cko^ cells (Figure 4I). Western blot analysis demonstrated that the upregulation of ZBP1 protein required the simultaneous presence of both mechanical stretch and high extracellular Ca^2^^+^ (Figure 4J,K). Immunofluorescence analysis further demonstrated that mechanical stretch induced robust Z‐NA formation and its colocalization with ZBP1 only in the presence of extracellular Ca^2^^+^. In contrast, under Ca^2^^+^‐free conditions, stretch‐induced Z‐NA and ZBP1 signals were nearly abolished and remained comparable to unstretched controls regardless of Ca^2^^+^ availability (Figure 4L). Finally, in vitro osteoclast differentiation assays confirmed the necessity of ZBP1 in this pathway: Yoda1‐induced osteoclast differentiation was significantly attenuated in *Zbp1* ^cko^ BMDMs. Consistently, when cultured in Ca^2+^‐free medium, Yoda1‐induced osteoclast formation was impaired (Figure 4 M,N).

### 3.5 *Zbp1* overexpression restores OTM in *Piezo1*‐deficient mice

**FIGURE 5:**
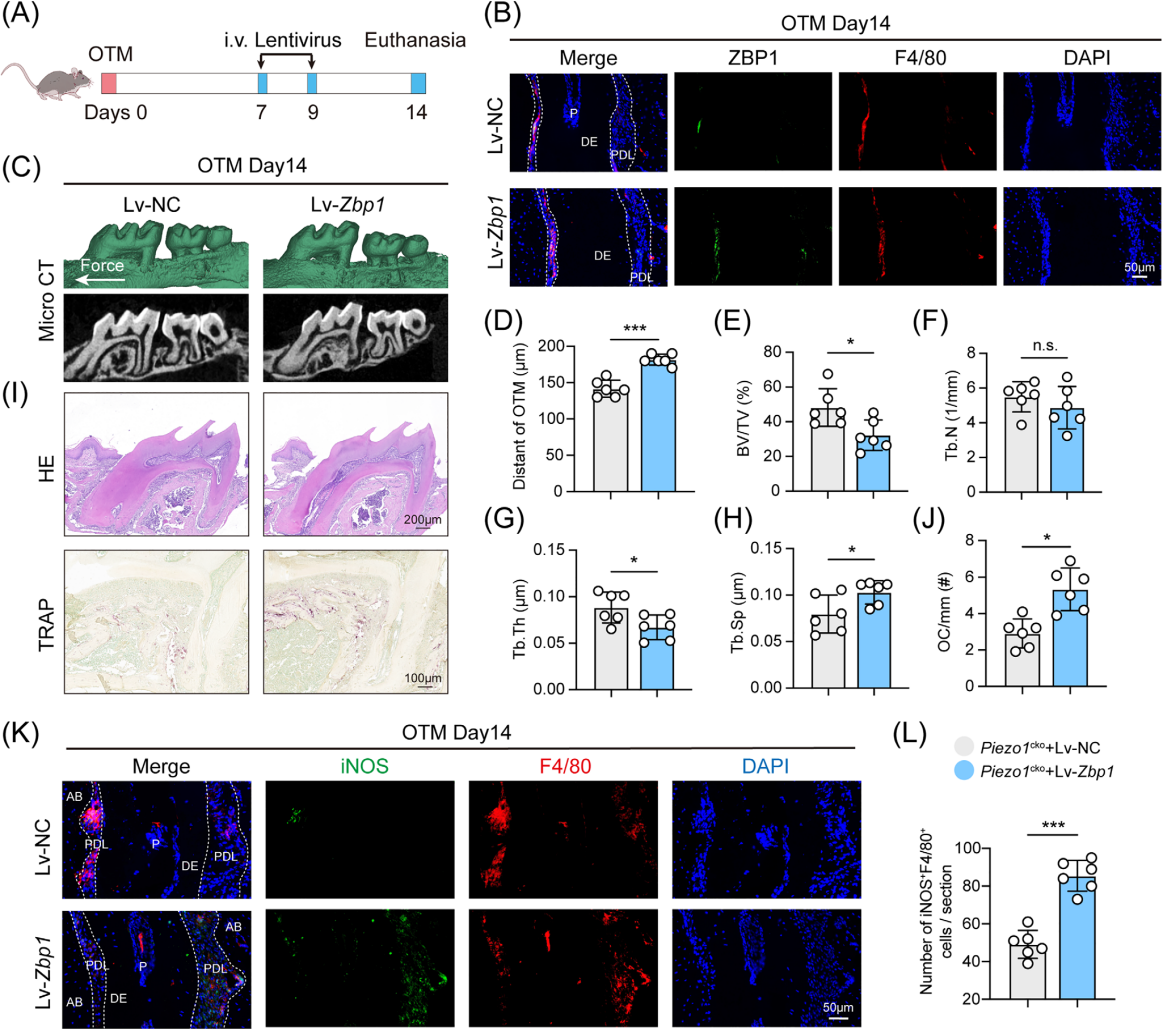
*Zbp1* overexpression restores OTM in *Piezo1*‐deficient mice. (A) Schematic timeline of *Zbp1*‐overexpressing lentivirus (Lv‐*Zbp1*) or control lentivirus (Lv‐NC) administration during the mouse OTM experiment. (B) Representative immunofluorescence images confirming *Zbp1* overexpression (green) in the periodontium of Lv‐*Zbp1* mice. Scale bar: 50 µm. (C and D) Representative three‐dimensional micro‐CT reconstructions of the maxillae from Lv‐NC and Lv‐*Zbp1* mice after 14 days of OTM and quantification of OTM distance (*n *= 6). (E–H) Micro‐CT analysis of alveolar bone microarchitecture on the tension side, including BV/TV, Tb.N, Tb.Th and Tb.Sp (*n *= 6). (I and J) Representative H&E and TRAP‐stained sections of the periodontium in Lv‐NC and Lv‐*Zbp1* mice after 14 days of OTM and quantification of osteoclast numbers per bone perimeter. Scale bar of H&E staining images: 200 µm; Scale bar of TRAP staining images: 100 µm (*n *= 6). (K) Representative immunofluorescence images of F4/80^+^iNOS^+^ cell infiltration in Lv‐NC and Lv‐*Zbp1* mice. AB, alveolar bone; PDL, periodontal ligament; DE, dentin; P, tooth pulp. Scale bar of immunofluorescence staining images: 50 µm. (L) Quantification of iNOS^+^F4/80^+^ cell numbers per section (*n *= 6). All data are presented as mean ± SD (*p < 0.05, **p < 0.01, ***p < 0.001).

To further investigate whether ZBP1 can rescue the slowed bone remodelling upon macrophage *Piezo1* knockout, *Piezo1^cko^* mice were administered *Zbp1*‐overexpressing lentivirus (Lv‐*Zbp1*) or control lentivirus (Lv‐NC) via the tail veil injection on Days 7, 9 and 11 following OTM induction (Figure 5A). Validation experiments confirmed efficient lentiviral‐mediated ZBP1 overexpression in stretched BMDMs, as shown by upregulated *Zbp1* mRNA and protein levels (Figure S6). Immunofluorescence staining confirmed successful ZBP1 protein overexpression in the periodontal tissues (Figure 5B).

Micro‐CT analysis at Day 14 revealed a significantly greater tooth movement distance in the Lv‐*Zbp1* group compared to the Lv‐NC controls (Figure 5C,D). Further bone morphometric analysis demonstrated that the *Zbp1*‐overexpressing mice exhibited significantly reduced BV/TV, Tb.N and Tb.Th, along with increased Tb.Sp on the tension side of the alveolar bone (Figure 5E–H). TRAP staining confirmed markedly enhanced bone resorption activity in the overexpression group, characterized by more osteoclasts along the bone surface (Figure 5I,J). Furthermore, the proportion of F4/80^+^iNOS^+^ macrophages was significantly higher in the Lv‐*Zbp1* group (Figure 5K,L). These findings collectively indicate that ZBP1 shapes a pro‐osteoclastic immune microenvironment and *Zbp1* overexpression restores OTM in *Piezo1*‐deficient mice.

### 3.6 Identification of JNJ‐10311795 as a ZBP1 inhibitor

**FIGURE 6:**
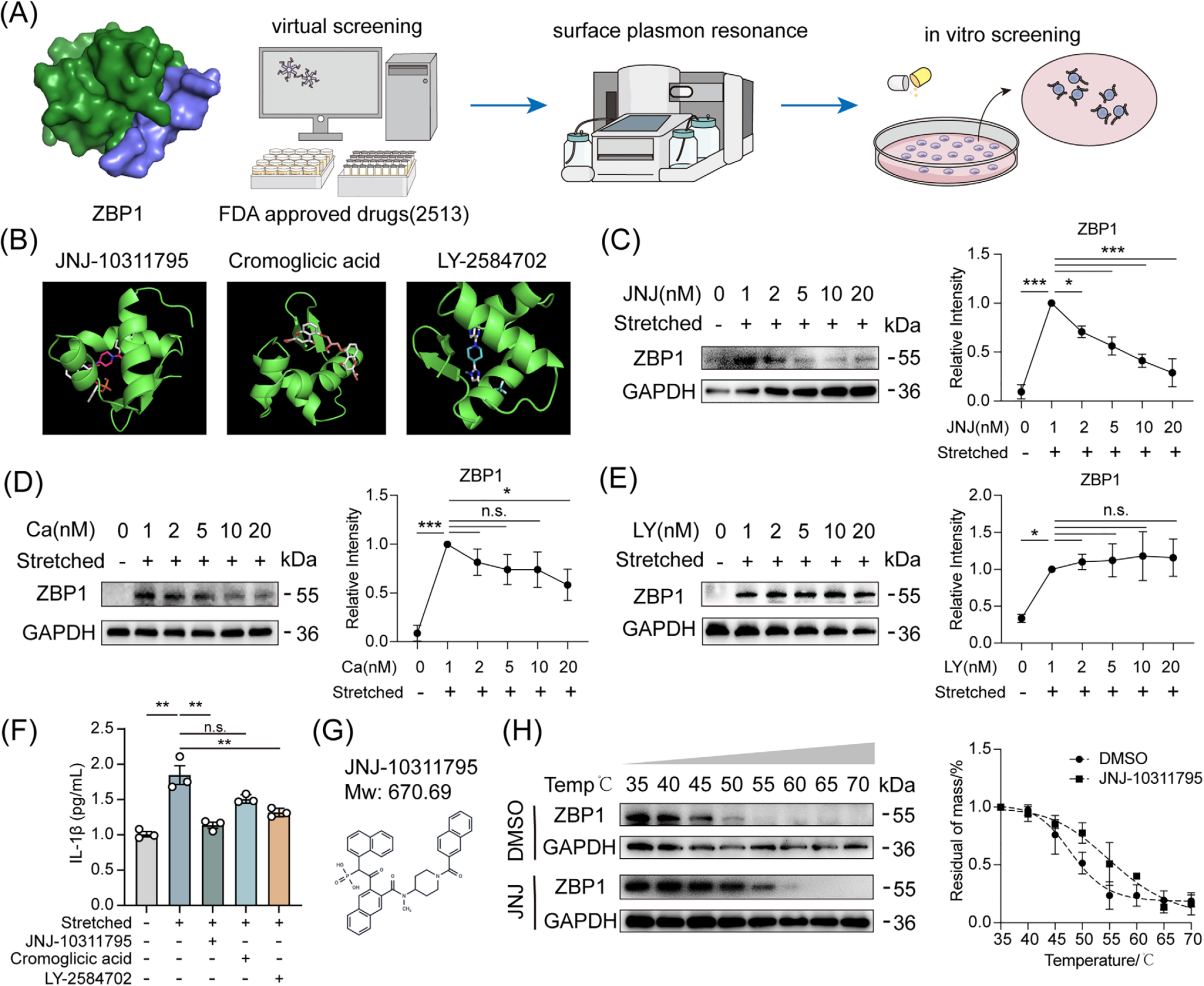
Identification and characterization of JNJ‐10311795 as a ZBP1 inhibitor. (A) Workflow of the structure‐based virtual screening for ZBP1 inhibitors. (B) Chemical structures of the top candidate ZBP1‐binding molecules identified from the screen. (C–E) Western blot analysis of ZBP1 protein expression in mechanically stimulated BMDMs treated with increasing doses of JNJ‐10311795, Cromoglicic acid and LY‐2584702 (0, 1, 2, 5, 10, 20 nM). (F) ELISA measurement of IL‐1β protein levels in the culture supernatant of mechanically stimulated BMDMs treated with or without JNJ‐10311795, Cromoglicic acid and LY‐2584702 (20 nM) (*n *= 3). (G) Chemical structure of the lead compound JNJ‐10311795. (H) Cellular Thermal Shift Assay (CETSA) demonstrating that JNJ‐10311795 interacts with ZBP1 protein, as indicated by the increased thermal stability of ZBP1 in JNJ‐10311795‐treated cells compared to DMSO controls. All data are presented as mean ± SD (*p < 0.05, **p < 0.01, ***p < 0.001).

To develop targeted therapeutic strategies against the Piezo1‐ZBP1 axis, we performed a structure‐based virtual screening targeting the Zα domain of ZBP1. The Zα domain crystal structure (PDB ID: 3EYI) was used as the docking receptor, and an FDA‐approved drug library containing 2513 compounds was screened. Compounds were ranked by predicted binding free energy (ΔG), and top candidates were further filtered by structural diversity, drug‐likeness and commercial availability. This approach identified JNJ‐10311795, Cromoglicic acid and LY‐2584702 as candidate ZBP1‐binding molecules (Figure 6A,B).

We next evaluated the functional activity of these three candidates in mechanically stimulated BMDMs. Western blot analysis revealed that both JNJ‐10311795 and Cromoglicic acid dose‐dependently reduced ZBP1 protein levels, with JNJ‐10311795 exhibiting more potent inhibitory effects. In contrast, LY‐2584702 showed no inhibition of ZBP1 expression across the tested concentration range (Figure 6C–E). We then examined whether these compounds could block force‐induced IL‐1β upregulation, a key pro‐inflammatory cytokine downstream of ZBP1 activation. Interestingly, both JNJ‐10311795 and LY‐2584702 significantly reduced IL‐1β levels, whereas Cromoglicic acid showed no significant effect (Figure 6F). Given that LY‐2584702 suppressed IL‐1β without substantially reducing ZBP1 expression, its anti‐inflammatory effect may involve off‐target mechanisms rather than direct ZBP1 modulation. Based on its superior efficacy in both reducing ZBP1 levels and blocking the downstream inflammatory response, JNJ‐10311795 was selected as the lead compound for further validation (structure shown in Figure 6G).

To further characterize the interaction between JNJ‐10311795 and ZBP1 in intact cells, we performed a CETSA. Mechanistically, if a compound engages its target protein within cells, it often enhances the protein's resistance to thermal denaturation. BMDMs were treated with JNJ‐10311795 or DMSO, subjected to mechanical stretch, then exposed to a temperature gradient (35°C–70°C). The soluble fraction of ZBP1 was assessed by Western blot. JNJ‐10311795 treatment significantly increased the thermal stability of ZBP1 compared to DMSO controls (Figure 6H), indicating that JNJ‐10311795 engages ZBP1 within the cellular environment and stabilizes the protein against heat‐induced aggregation. Collectively, these in vitro results establish JNJ‐10311795 as a potent ZBP1 modulator capable of suppressing Piezo1‐induced ZBP1 expression and downstream inflammation in macrophages.

### 3.7 JNJ‐10311795 attenuates OTM and modifies fracture healing

**FIGURE 7:**
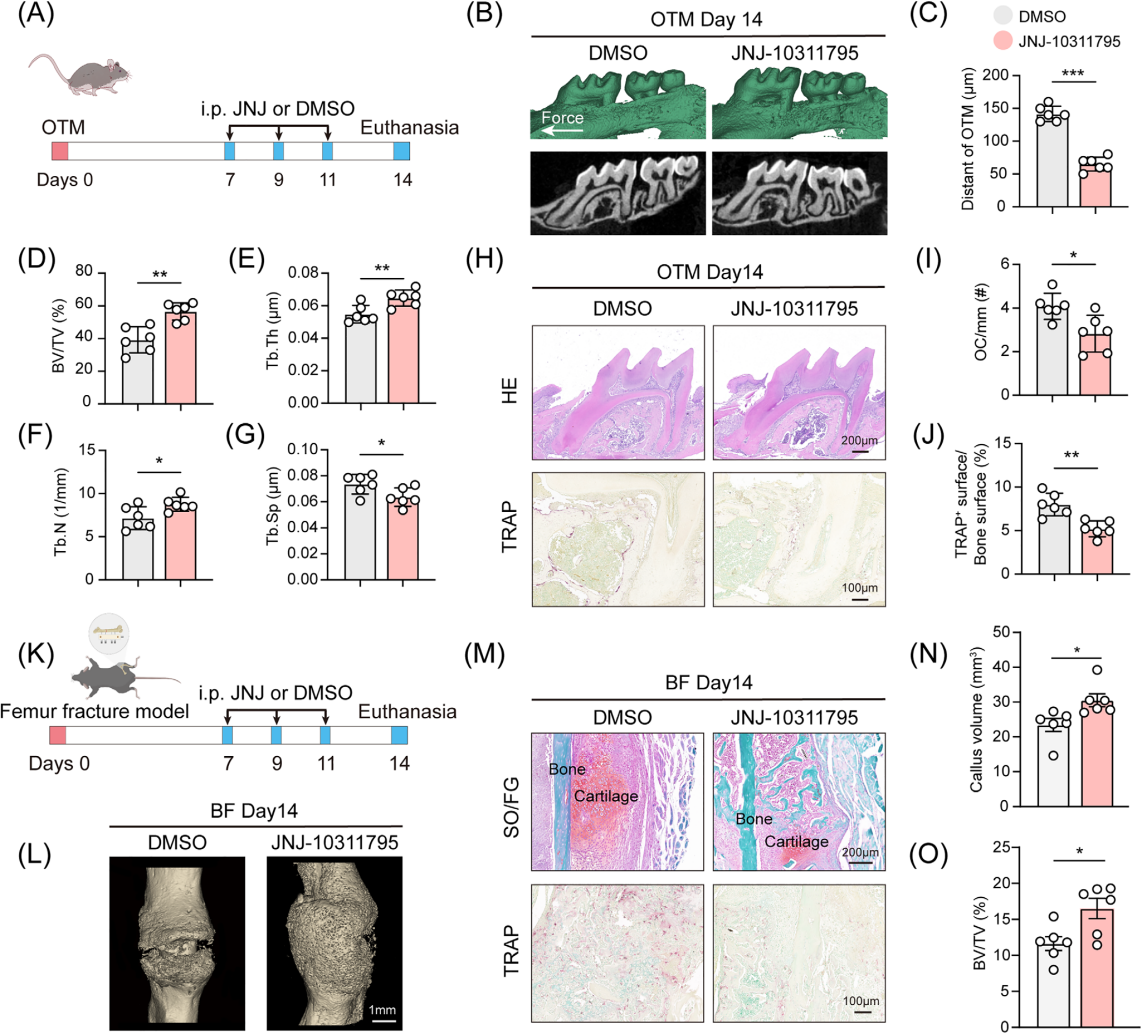
ZBP1 inhibitor JNJ‐10311795 attenuates OTM and modulates bone fracture healing. (A) Schematic timeline of JNJ‐10311795 (1 mg/kg) or vehicle (DMSO) administration during the mouse OTM experiment. (B and C) Representative micro‐CT reconstructions and quantification of OTM distance in vehicle and JNJ‐10311795‐treated mice at day 14 (*n *= 6). (D–G) Micro‐CT analysis of alveolar bone microarchitecture on the tension side, including BV/TV, Tb.Th, Tb.N, and Tb.Sp (*n *= 6). (H) Representative H&E and TRAP‐stained sections of the periodontium in vehicle and JNJ‐10311795‐treated mice after 14 days of OTM. Scale bar of H&E staining images: 200 µm; Scale bar of TRAP staining images: 100 µm. (I and J) Quantification of osteoclast numbers per bone perimeter and TRAP‐positive surface relative to the bone surface (*n *= 6). (K) Schematic diagram of the mouse femur fracture model and schematic timeline of JNJ‐10311795 (1 mg/kg) or vehicle (DMSO) administration during the mouse fracture healing experiment. (L and N) Representative micro‐CT reconstructions of femoral fracture callus and quantification of callus volume in vehicle and JNJ‐10311795‐treated mice (*n *= 6). (M) Representative safranin O/fast green‐ and TRAP‐stained sections of the femoral fracture callus in vehicle and JNJ‐10311795‐treated mice. Scale bar of safranin O/fast green staining images: 200 µm; Scale bar of TRAP staining images: 100 µm. (O) Micro‐CT analysis of fracture callus microarchitecture (BV/TV) (*n *= 6). All data are presented as mean ± SD (*p < 0.05, **p < 0.01).

To evaluate the therapeutic potential of JNJ‐10311795 in vivo, we employed two distinct mouse models: a 14‐day OTM model and a femoral fracture healing model. In the OTM experiments, JNJ‐10311795 (1 mg/kg) or vehicle (DMSO) was administered via intraperitoneal injection on Days 7, 9, and 11 after force application (Figure 7A). Before assessing whole‐animal phenotypes, we first validated target engagement in periodontal tissues. qPCR and Western blot analysis confirmed that JNJ‐10311795 treatment significantly reduced ZBP1 in the periodontium of wild‐type mice under orthodontic force (Figure S7), demonstrating effective in vivo target modulation.

Following this local tissue validation, we examined the functional outcomes. Micro‐CT analysis at Day 14 revealed that JNJ‐10311795 treatment significantly reduced tooth movement distance compared to the DMSO control group (Figure 7B,C). Bone microstructure analysis on the tension side showed that JNJ‐10311795 treatment significantly improved BV/TV, Tb.Th, and Tb.N, while reducing Tb.Sp (Figure 7D–G). Histology confirmed that the tissue remodelling process of the JNJ‐10311795 treatment group was weakened, and bone resorption was inhibited. TRAP staining further showed that the number of osteoclasts on the pressure side of the JNJ‐10311795 treatment group was significantly reduced (Figure 7H–J). Genetic epistasis experiments further demonstrated that JNJ‐10311795 significantly reduced OTM distance and osteoclast number in *Zbp1* ^f/f^ mice, but these effects were completely absent in *Zbp1* ^cko^ mice (Figure S8), confirming that the inhibitory action of JNJ‐10311795 on tooth movement is ZBP1‐dependent.

To assess broader therapeutic implications, we next examined fracture healing under ZBP1 inhibition using the same dosing regimen (Figure 7K). Micro‐CT analysis showed that JNJ‐10311795‐treated mice formed a larger and denser callus, with significantly increased callus volume and BV/TV compared to controls (Figure 7L,N,O). The SO/FG staining at Day 14 after the fracture showed that callus in the DMSO group was in the intermediate stage of endochondral ossification, while the JNJ‐10311795 group had formed a large number of solid green stained woven bone, suggesting that the repair process was more advanced (Figure 7 M). At the same time, TRAP staining showed that there were abundant osteoclasts in the callus area of the DMSO group, while this phenomenon was significantly reduced in the JNJ‐10311795 treatment group (Figure 7 M). Consistently, genetic epistasis experiments showed that JNJ‐10311795 increased callus volume and BV/TV only in *Zbp1* ^f/f^ mice, with no additional effect in *Zbp1* ^cko^ mice (Figure S9). These findings show that inhibiting ZBP1 can not only slow down OTM by reducing osteoclastogenesis, but also accelerate fracture repair by promoting the formation of woven bone and regulating endochondral ossification.

## 4 DISCUSSION

Mechanical cues regulate bone remodelling under a broad spectrum of physiological and pathological conditions, including OTM, fracture repair and mechanical load‐driven bone adaptation.^5^, ^10^ However, it remains unclear exactly how physical force triggers the intracellular signals that ultimately lead to bone resorption.^13^ In this study, we found that when macrophages sense mechanical force, they activate the Piezo1‐ZBP1 pathway, which in turn triggers inflammation and the formation of osteoclasts. The striking consistency between human and mouse data, spanning from gene expression patterns to cellular responses, provides strong confidence for the clinical translation of this pathway in both orthodontics and orthopaedics.

Clinical observations in orthodontic patients provided the initial evidence. Expression of *PIEZO1* and *ZBP1* in human periodontal ligament tissue was upregulated after orthodontic force loading, with a strong positive correlation between the two. Studies in mouse models confirmed that these findings are consistent across species. In mice, orthodontic force increased *Piezo1* and *Zbp1* levels in PDL macrophages, with the highest expression observed at 7 to 14 days, exactly when macrophages infiltrate the tissue and osteoclasts become most active. This concordance, together with correlation analyses showing positive links between *PIEZO1*/*ZBP1* and pro‐inflammatory cytokines in human samples, suggests that this axis operates in human mechanotransduction.

Genetic loss‐of‐function experiments confirmed that this signalling axis is functionally essential. Macrophage‐specific deletion of either *Piezo1* or *Zbp1* significantly slowed OTM, reduced osteoclast formation, and preserved alveolar bone mass. Conversely, overexpressing *Zbp1* in *Piezo1*‐deficient mice rescued the impaired OTM phenotype, demonstrating that Piezo1 acts upstream of ZBP1 in this pathway. Mechanistically, Piezo1‐mediated Ca^2^^+^ influx was both necessary and sufficient for ZBP1 upregulation and subsequent osteoclast formation, as demonstrated by Ca^2^^+^‐free conditions and Yoda1 stimulation.

Importantly, the relevance of this axis extends beyond OTM. In a femoral fracture healing model, which represents a fundamentally different mechanical and inflammatory environment, macrophage‐specific *Piezo1* deletion similarly accelerated bone repair, as evidenced by enhanced callus formation and woven bone deposition. Thus, the Piezo1‐ZBP1 axis operates in both OTM and fracture healing, suggesting that macrophages use this pathway as a general mechanism to sense mechanical stress and regulate bone remodelling.

The role of Piezo1 in bone biology is highly context‐dependent. In osteoblasts, Piezo1, as the core mechanical receptor of anabolic load, promotes bone formation^41^, ^42^, ^43^ through pathways such as Akt/GSK‐3β/β‐catenin and YAP/TAZ; its knockout impairs bone formation, and via the RANKL/OPG axis indirectly affects the activity of osteoclasts.^44^, ^45^ In non‐inflammatory regenerative settings, macrophage Piezo1 supports a pro‐repair M2 phenotype, maintaining tissue homeostasis and osteogenesis.^21^, ^46^ In contrast, our study reveals that the macrophage Piezo1 has distinct pro‐inflammatory and bone‐remodelling functions in the specific inflammatory microenvironment of OTM. This aligns with emerging evidence that, under conditions of pathological or excessive mechanical stress (e.g., periodontitis or heavy orthodontic force), the activation of Piezo1 in periodontal cells or macrophages drives pro‐inflammatory M1 polarization and associated tissue damage.^17^, ^23^ Similarly, in a bone infection model, Piezo1 was found to promote the polarization of M1 macrophages and impair osteogenesis.^47^ The functional divergence of macrophage Piezo1 likely arises from the integration of disparate microenvironmental cues: in sterile regeneration, mechanical cues may promote M2 polarization, whereas in injury‐associated inflammatory settings like OTM, mechanical signals may synergize with damage‐associated molecular patterns^48^ to drive a pro‐inflammatory, pro‐remodelling M1 phenotype. Our use of macrophage‐specific genetic knockout models specifically clarifies the contribution of macrophage‐intrinsic Piezo1 signalling to this process, isolating it from potential compensatory or opposing effects from other cell types. Other mechanosensitive channels, including TRPV4, TRPM7 and TREK‐1, may also contribute to these processes, though their relative importance remains to be determined.^49^, ^50^, ^51^

Conceptually, our work redefines ZBP1 from a canonical viral nucleic acid sensor to a novel effector in sterile mechanotransduction. We establish ZBP1 as a key downstream component of the Piezo1‐mediated Ca^2^^+^ signalling pathway, crucial for establishing an efficient osteoclastogenic microenvironment. To our knowledge, this is the first report linking ZBP1 to Piezo1‐dependent mechanobiology. The function of ZBP1 is highly context‐dependent: in viral infection, it is essential for host defence by inducing necroptosis;^26^ in the sepsis model, it promotes M1 polarization and related organ damage;^52^, ^53^ in the lung cancer, it inhibits M2 polarization;^54^ and in sterile inflammatory conditions such as OTM, it drives bone‐resorptive inflammation. Notably, in fracture healing, ZBP1‐driven inflammatory resorption helps clear damaged tissue, but excessive or sustained ZBP1 activity delays the transition to bone formation; consequently, ZBP1 inhibition accelerates healing by preventing this detrimental over‐resorption. These observations highlight the importance of timing and context for therapeutic targeting of ZBP1.

The association between Piezo1‐mediated Ca^2^^+^ influx and ZBP1 activation is supported by evidence across multiple disease models. Studies on acute kidney injury, sporotrichosis, and acute glaucoma indicate that disruption of calcium homeostasis is a key upstream trigger for ZBP1 upregulation and downstream PANoptosis.^55^, ^56^, ^57^, ^58^ The acute high intraocular pressure model directly confirms that mechanical force can induce calcium homeostasis disruption, driving ZBP1‐mediated cell death.^58^ Our data extend this mechanism paradigm to mechanotransduction and bone biology, establishing that the Piezo1‐Ca^2^^+^‐ZBP1 axis converts mechanical or physicochemical stress into innate immune and cellular responses.

While our data confirm that Piezo1‐mediated Ca^2^^+^ influx is required for ZBP1 upregulation, the specific transcription factors linking Ca^2^^+^ signalling to *Zbp1* promoter activation remain unidentified, with NFAT and AP‐1 as prime candidates.^59^, ^60^, ^61^ It also remains unclear whether the Piezo1‐ZBP1 axis drives osteoclastogenesis directly or indirectly via macrophage PANoptosis and subsequent DAMP release.^24^, ^55^ Finally, while our human data show cross‐species consistency, they are correlative and await functional validation in primary human macrophages. Addressing these questions will require further investigation, including ChIP‐seq, promoter‐reporter assays, and orthogonal cell death assays.

Through structure‐based virtual screening, we identified JNJ‐10311795 as a ZBP1 modulator. CETSA confirmed that JNJ‐10311795 engages ZBP1 in intact cells. Strikingly, this compound inhibits ZBP1‐driven osteoclastogenesis, which translates into context‐dependent therapeutic outcomes: it attenuates excessive bone resorption during OTM, while in fracture healing, curbing the resorption of cartilaginous callus promotes net bone formation and accelerates repair. Several non‐mutually exclusive mechanisms may explain its pro‐osteogenic effect: reduction of pro‐inflammatory cytokines that inhibit osteoblast differentiation, preservation of the cartilaginous callus template allowing orderly endochondral ossification, and alteration of the macrophage secretome to promote osteoblast recruitment. Genetic epistasis experiments showed that JNJ‐10311795 had no additional effects in *Zbp1* ^cko^ mice in either model, strongly supporting that its in vivo actions are mediated through ZBP1 rather than off‐target pathways. Nevertheless, we acknowledge that JNJ‐10311795 is also a known dual inhibitor of chymase and cathepsin G,^62^, ^63^ and its multi‐target nature may contribute to its overall efficacy; future studies using more selective ZBP1 inhibitors will help to further clarify the specific contribution of targeting ZBP1.

From the perspective of clinical orthodontics, the ability to pharmacologically decelerate OTM provides a novel ‘molecular brake’ for higher‐precision treatment. Critically, JNJ‐10311795 is suitable for localized administration, opening possibilities for ‘regional anchorage enhancement’. Several administration strategies can be considered: local injection into the periodontal ligament space; encapsulation in biodegradable hydrogels or electrospun membranes for sustained release; or coating onto orthodontic appliances or micro‐implants for site‐specific delivery. However, the optimal formulation, dosing regimen, and long‐term safety need systematic evaluation in animal models before clinical application. Beyond orthodontics, this signalling axis represents a promising therapeutic target for a spectrum of skeletal disorders characterized by imbalanced bone remodelling, ranging from periodontal disease to fracture non‐union or osteolytic lesions.

The discovery of the Piezo1‐ZBP1 axis opens promising avenues for future investigation. Key aspects include elucidating how Piezo1‐mediated Ca^2^^+^ influx regulates *Zbp1* gene transcription, where calcineurin‐NFAT may play a role, and identifying the endogenous ligand that activates ZBP1 under sterile mechanical stress. We speculate that mechanical force may induce production of endogenous Z‐form nucleic acids, such as mitochondrial DNA or retroelement transcripts, which could act as physiological ligands to activate ZBP1. It would also be worthwhile to investigate the potential involvement of ZBP1's classical interaction proteins (e.g., RIPK1/RIPK3) or its nucleic acid perception function in this sterile mechanotransduction.

**FIGURE 8:**
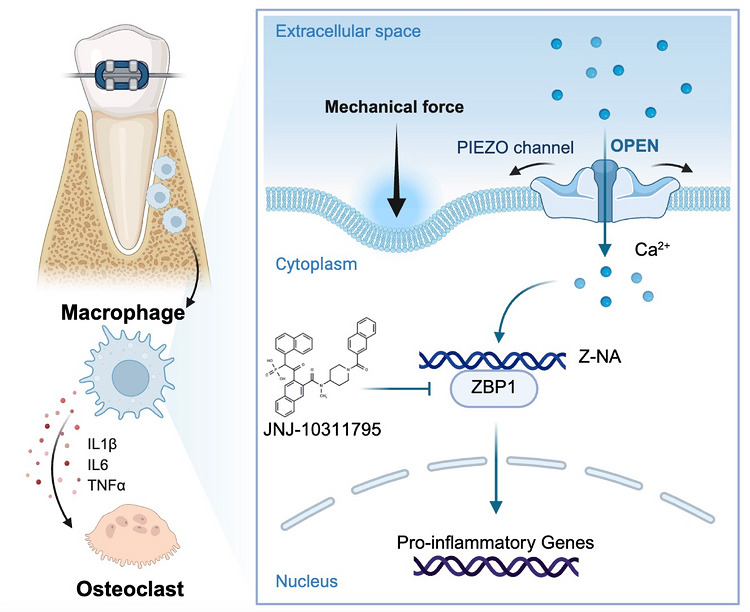
Graphic abstract of this study. In the periodontal microenvironment, orthodontic mechanical force activates the mechanosensitive ion channel Piezo1 on macrophages, triggering Ca^2+^ influx. The increased intracellular Ca^2+^ concentration drives the upregulation and activation of the innate immune sensor ZBP1, promoting the formation of Z‐NA. Activated ZBP1 initiates a pro‐inflammatory response, leading to the enhanced transcription and release of cytokines such as IL‐1β, IL‐6 and TNF‐α. This inflammatory milieu, along with direct signalling, promotes osteoclast differentiation and activation, thereby driving alveolar bone resorption essential for orthodontic tooth movement. The novel small‐molecule compound JNJ‐10311795, identified as a ZBP1 inhibitor, blocks this pathway, attenuating inflammation and osteoclastogenesis. This schematic was created with BioRender (https://biorender.com).

In conclusion, our work defines the macrophage Piezo1‐ZBP1 signalling axis as a fundamental mechano‐immune switch that translates mechanical force into inflammatory bone remodelling (Figure 8). This mechanism operates across both OTM and fracture healing, where controlled bone resorption is required for tissue remodelling. Validated through integrated genetic and pharmacological approaches across multiple in vivo and in vitro models, the axis provides a unified therapeutic target for intervening in pathological bone remodelling in a variety of skeletal disorders.

## AUTHOR CONTRIBUTIONS

**Linlin Zhu**: Methodology, investigation, writing—original draft, review, and editing. **Kejia Zhang**: Methodology, investigation, writing—original draft, review, and editing. **An Song**: Methodology, investigation. **Weiye Zhao**: Investigation, writing–review. **Yongze Lin**: Investigation. **Hao Xu**: Resources, supervision. **Zhichun Jin**: Resources, supervision. **Yan Wang**: Investigation. **Shengnan Wu**: Investigation. **Xueyu Sun**: Investigation. **HengGu**: Investigation. **Ming Lu**: Methodology, supervision, writing—review and editing. **Hanwen Zhang**: Methodology, supervision, writing—review and editing. **Bin Yan**: Conceptualisation, methodology, writing—review and editing, funding acquisition. All authors reviewed the manuscript and approved its final version.

## CONFLICT OF INTEREST STATEMENT

The authors declare no conflicts of interest.

## ETHICS STATEMENT

This study involving human periodontal ligament (PDL) samples collected from orthodontic patients under informed consent (approved by the Ethics Committee of the Affiliated Stomatological Hospital of Nanjing Medical University, No. PJ2021‐058‐01) and animal experiments on mice (approved by the Institutional Animal Care and Use Committee of Nanjing Medical University, Nos. IACUC‐2309028 and IACUC‐2503016) was conducted in strict adherence to the Declaration of Helsinki and institutional guidelines. All procedures involving human participants and animals were provided in the *Materials and Methods* section.

## Supporting information



Supporting Information

Supporting Information

Supporting Information

## Data Availability

The RNA‐seq data of macrophages (*Piezo1* ^f/f^ and *Piezo1* ^cko^) were deposited in Genome Sequence Archive database (https://ngdc.cncb.ac.cn/gsub/) with accession number PRJCA057077 (https://ngdc.cncb.ac.cn/bioproject/browse/PRJCA057077). All data are included within the article or Supporting Information are available from the authors on request.
