# Supplementary material for: Macrophage piezo1 senses mechanical force to drive osteoclastogenesis via ZBP1: Implications for bone remodelling therapy
Source: Clin Transl Med. 2026 May 23;16(5):e70703. doi: 10.1002/ctm2.70703 (PMC13239690; doi:10.1002/ctm2.70703)
Supplement: Supplementary file 2 — Supporting Information [file CTM2-16-e70703-s002.docx]

**Supplementary materials**

**Supplemental Figures**

**Figure S1**


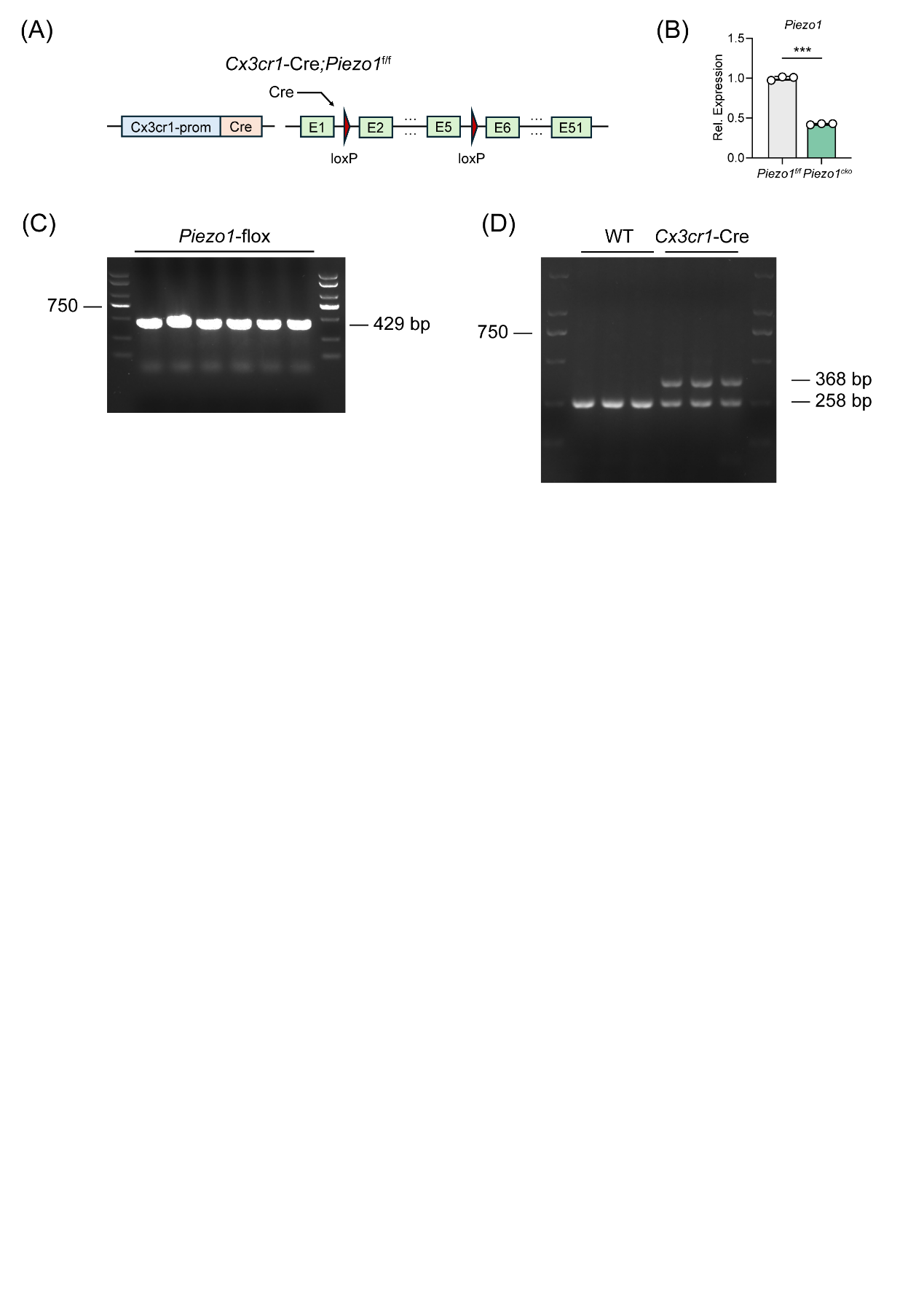


**Figure S1. Generation of Macrophage-specific *Piezo1* knockout mice.**

(A) Illustration of *Piezo1* deletion in *Cx3cr1*-expressing macrophages. (B) mRNA expression of *Piezo1* in BMDMs of *Piezo1^f/f^* and *Piezo1^cko^* mice was measured by qPCR (*n*=3). (C) Gel electrophoresis for *Piezo1*-flox genotyping. (D) Gel electrophoresis for *Cx3cr1*-cre genotyping. All data are presented as mean ± SD (****P* < 0.001).

**Figure S2**


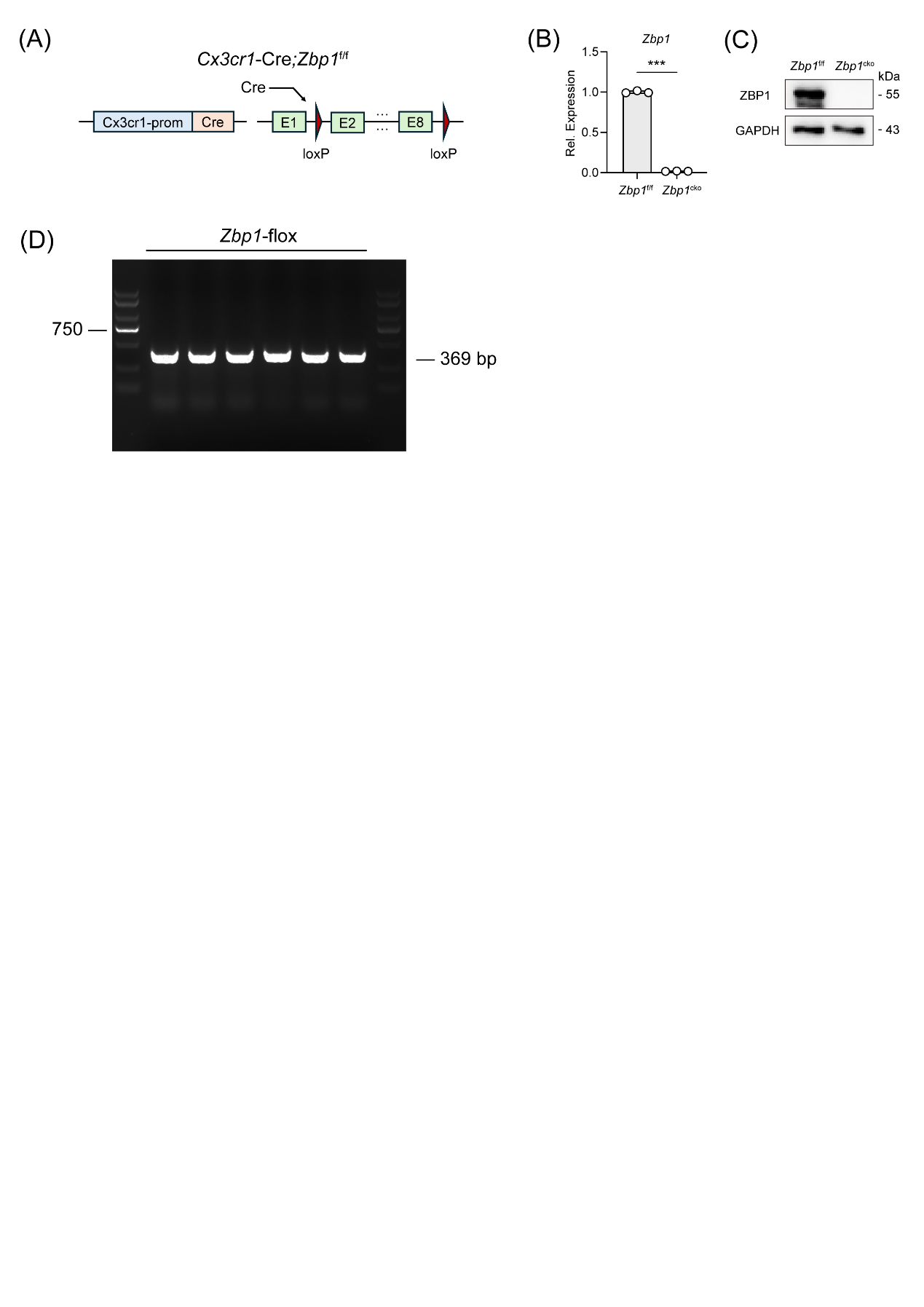


**Figure S2. Generation of Macrophage-specific *Zbp1* knockout mice.**

(A) Illustration of *Zbp1* deletion in *Cx3cr1*-expressing macrophages. (B) mRNA expression of *Zbp1* in BMDMs of *Zbp1*^f/f^ and *Zbp1*^cko^ mice was measured by qPCR (*n* = 3). (C) Western blot validation of *Zbp1* knockout efficiency. (D) Gel electrophoresis for *Piezo1*-flox genotyping. All data are presented as mean ± SD (****P* < 0.001).

**Figure S3**

**
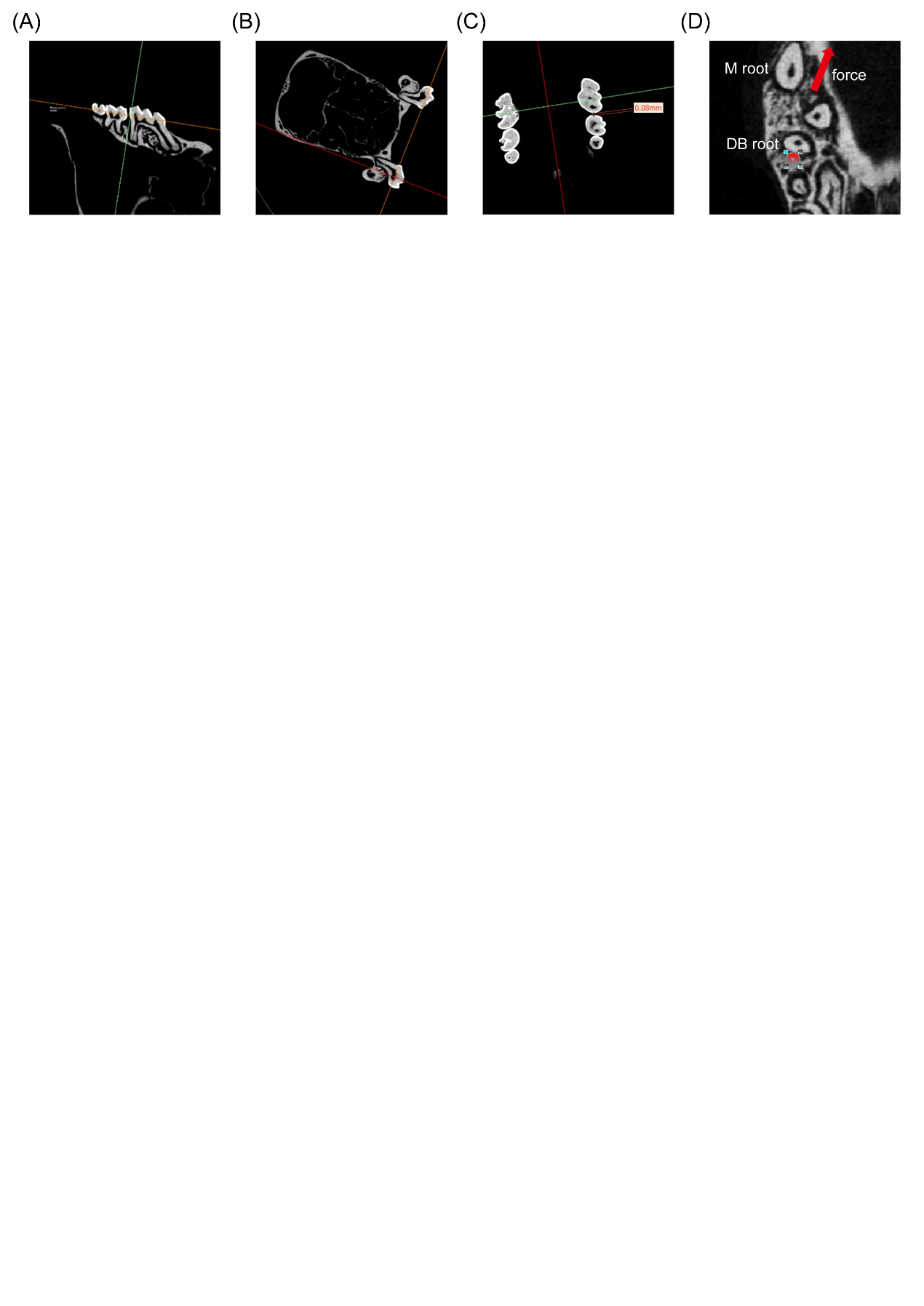
**

**Figure S3. Schematic diagram of Micro-CT measurement in mice.**

(A-C) Micro-CT measurement of OTM distance. ((A) sagittal plane, (B) coronal plane, (C) horizontal plane). (D) Micro-CT analysis of periradicular bone morphometry.

**Figure S4**


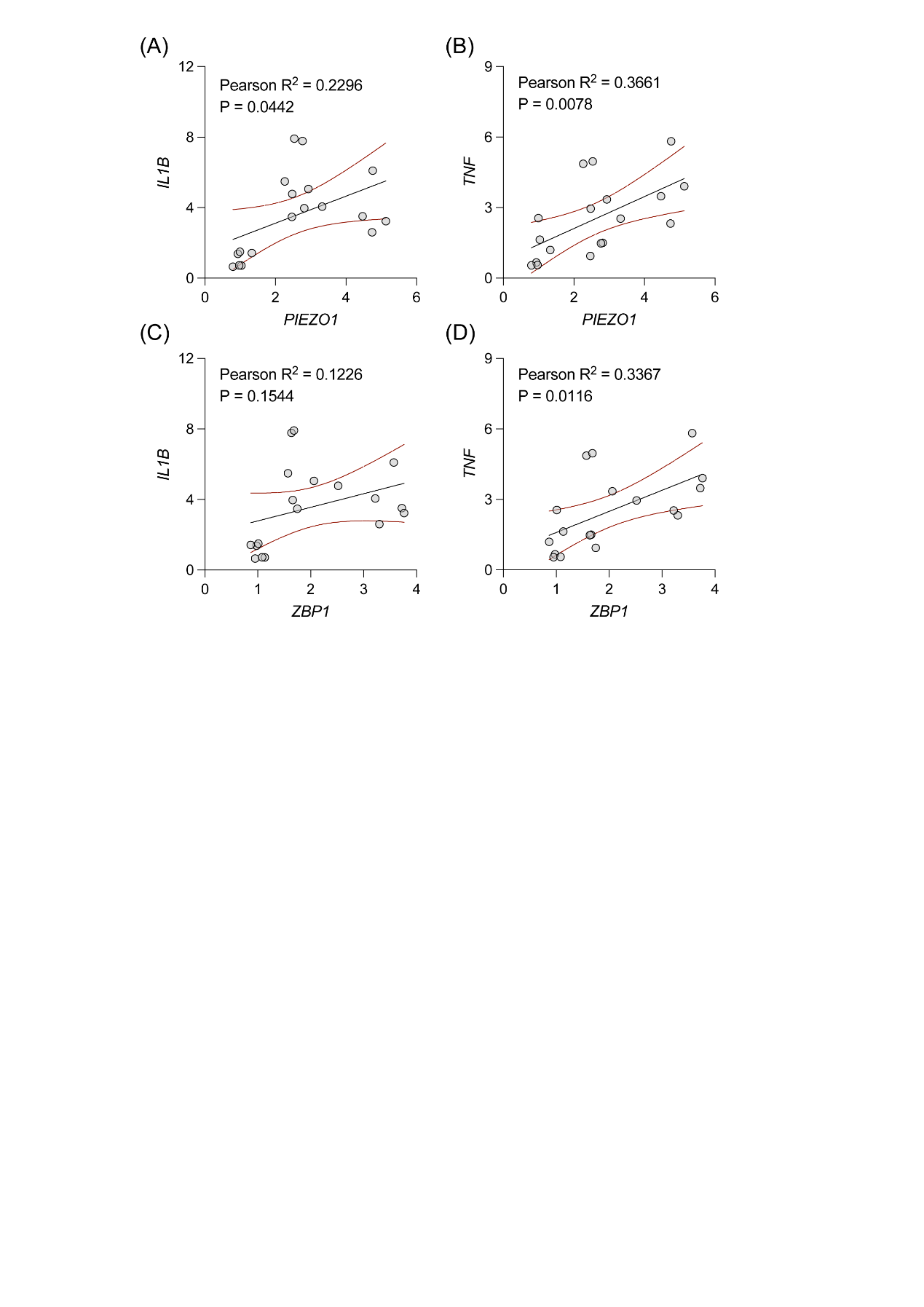


**Figure S4. Correlation analyses of mechanosensitive genes and pro‑inflammatory cytokines in human periodontal ligament (PDL) tissues under orthodontic force.**

(A) Spearman correlation analysis between *PIEZO1* and *IL1B* mRNA expression levels in human PDL samples (*n*=18). (B) Spearman correlation analysis between *PIEZO1* and *TNF* mRNA expression levels in human PDL samples (*n* =18). (C) Spearman correlation analysis between *ZBP1* and *IL1B* mRNA expression levels in human PDL samples (*n*=18). (D) Spearman correlation analysis between *ZBP1* and *TNF* mRNA expression levels in human PDL samples (*n*=18). Each dot represents one sample. Correlation coefficient (R) and P value are shown in each panel.

**Figure S5**


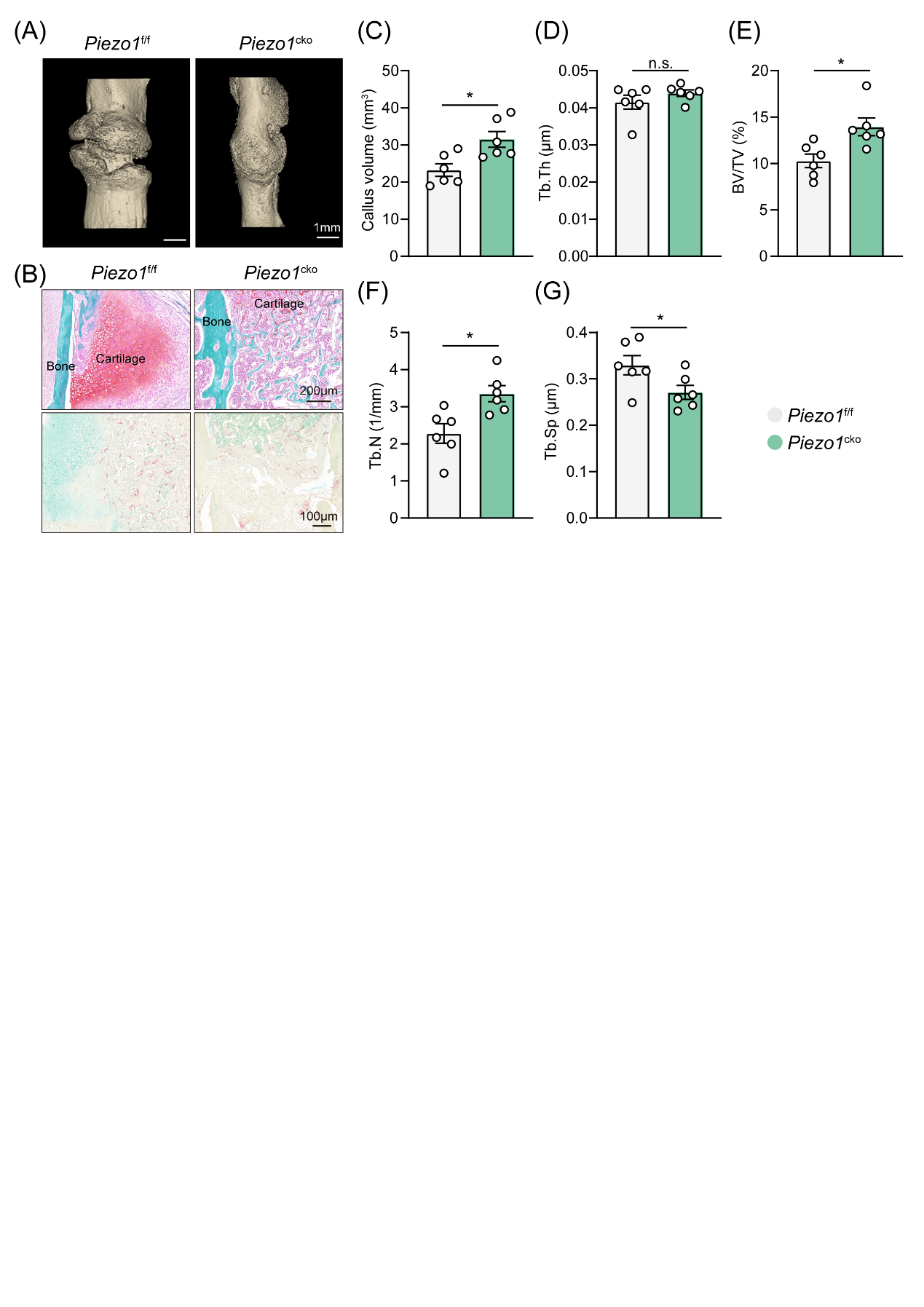


**Figure S5. Macrophage-specific *Piezo1* deletion alters fracture healing pattern.**

(A) Representative micro-CT reconstructions of femoral fracture callus in *Piezo1*^f/f^ and *Piezo1*^cko^ mice at the healing endpoint (*n*=6). (B) Representative safranin O/fast green- and TRAP-stained sections of the femoral fracture callus in *Piezo1*^f/f^ and *Piezo1*^cko^ mice. Scale bar of safranin O/fast green staining images, 200 μm; Scale bar of TRAP staining images, 100 μm. (C) Quantification of callus volume in *Piezo1*^f/f^ and *Piezo1*^cko^ mice at the healing endpoint (*n*=6). (D-G) Micro-CT analysis of fracture callus microarchitecture, including Tb.Th, BV/TV, Tb.N, and Tb.Sp (*n*=6). All data are presented as mean ± SD (n.s., no significance, **P* < 0.05).

**Figure S6**


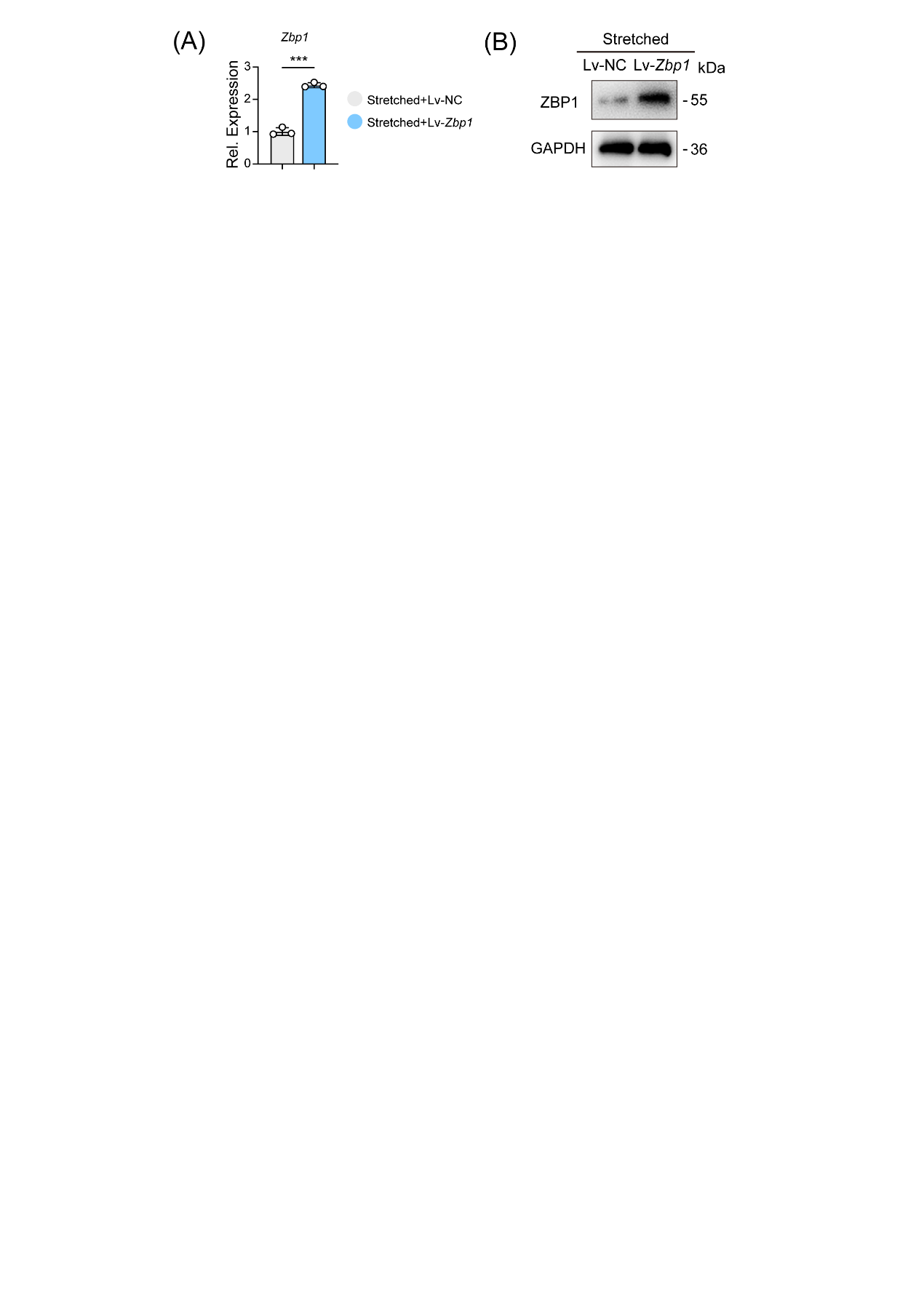


**Figure S6. Validation of lentiviral-mediated Zbp1 overexpression efficiency *in vitro*.**

(A) qPCR analysis showing significantly increased *Zbp1* mRNA levels in stretched BMDMs transduced with Lv-*Zbp1*, compared with the Lv-NC control (*n*=3). (B) Western blot analysis confirming significantly elevated ZBP1 protein expression in the Lv-*Zbp1*-transduced BMDMs under stretched conditions. GAPDH was used as a loading control. All data are presented as mean ± SD (****P* < 0.001).

**Figure S7**


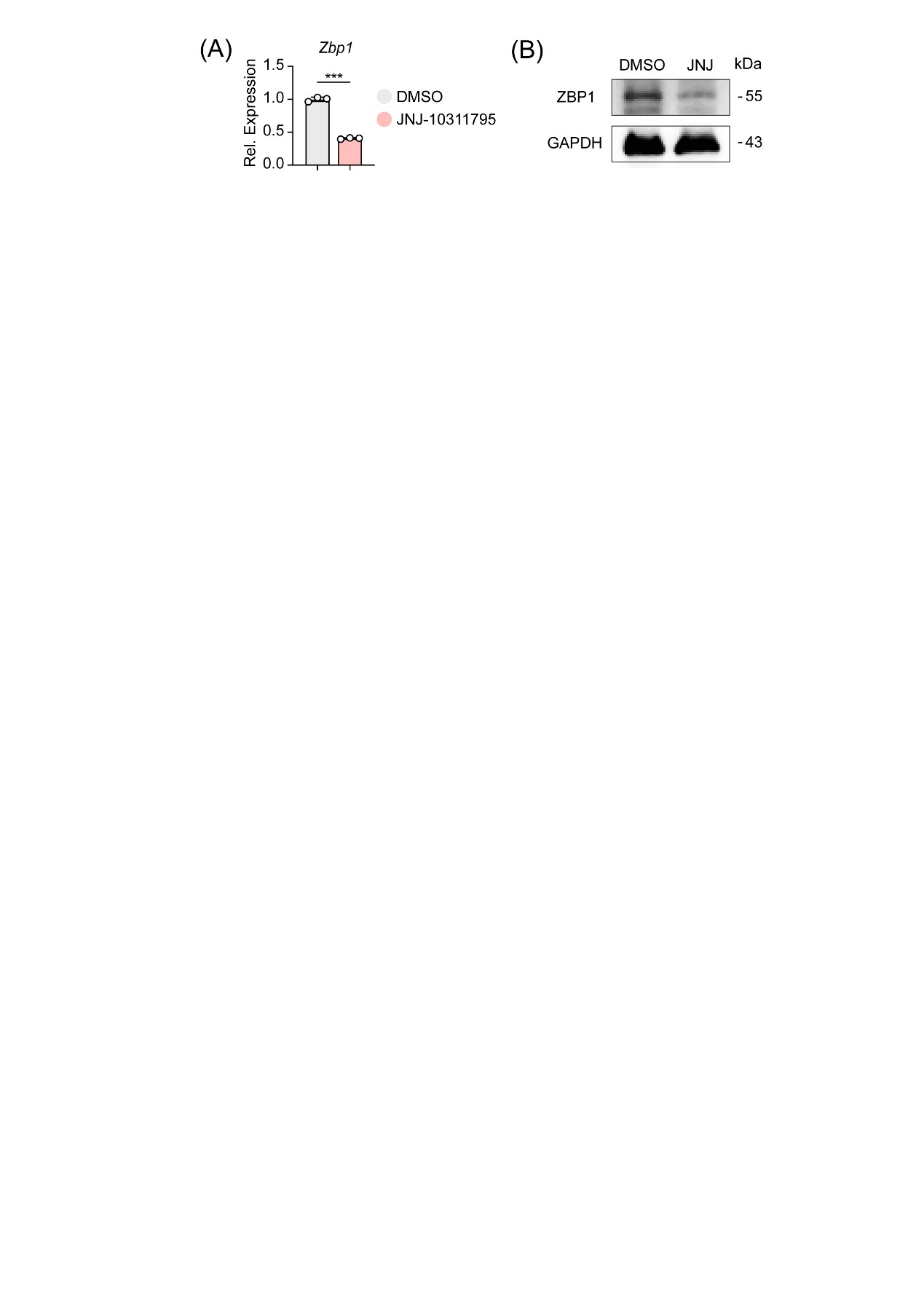


**Figure S7. JNJ-10311795 inhibits force-induced ZBP1 level in mouse periodontal tissues *in vivo*.**

(A) qPCR analysis showing that JNJ-10311795 treatment (1 mg/kg, i.p. on days 7, 9, 11) significantly reduced *Zbp1* mRNA expression in periodontal tissues of wild-type mice under orthodontic force (14 d OTM), compared with DMSO control (*n*=3). (B) Western blot analysis confirming that JNJ-10311795 treatment significantly inhibited ZBP1 protein expression in periodontal tissues of OTM mice. GAPDH was used as a loading control. All data are presented as mean ± SD (*P* < 0.001).

**Figure S8**


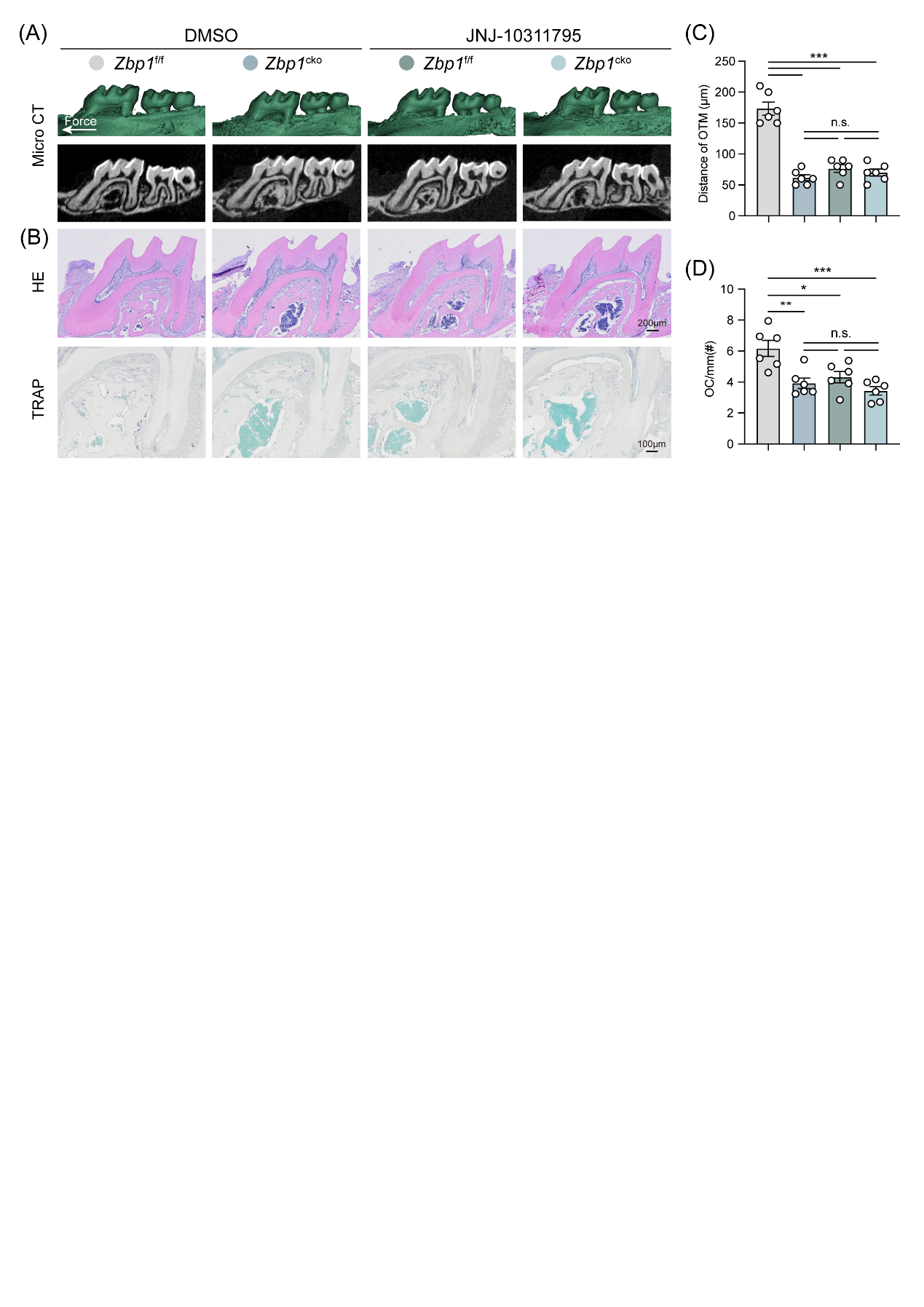


**Figure S8. JNJ-10311795 inhibits OTM by targeting *Zbp1* in macrophages.**

(A) Representative three-dimensional micro-CT reconstructions of the maxillae from *Zbp1*^f/f^ and *Zbp1*^cko^ mice treated with DMSO or JNJ-10311795 after 14 days of OTM. (B) Representative H&E and TRAP-stained sections of the periodontium in the indicated groups. Scale bar of H&E staining images, 200 μm; Scale bar of TRAP staining images, 100 μm. (C) Quantification of OTM distance in the indicated groups (*n*=6). JNJ-10311795 significantly reduced OTM distance in *Zbp1*^f/f^ mice, but this effect was abolished in *Zbp1*^cko^ mice. (D) Quantification of osteoclast numbers per bone perimeter (OC/mm) in the indicated groups (*n*=6). JNJ-10311795 significantly reduced osteoclast formation in *Zbp1*^f/f^ mice, but had no significant effect in *Zbp1*^cko^ mice. All data are presented as mean ± SD (n.s., no significance, **P* < 0.05, ***P* < 0.01, ****P* < 0.001).

**Figure S9**


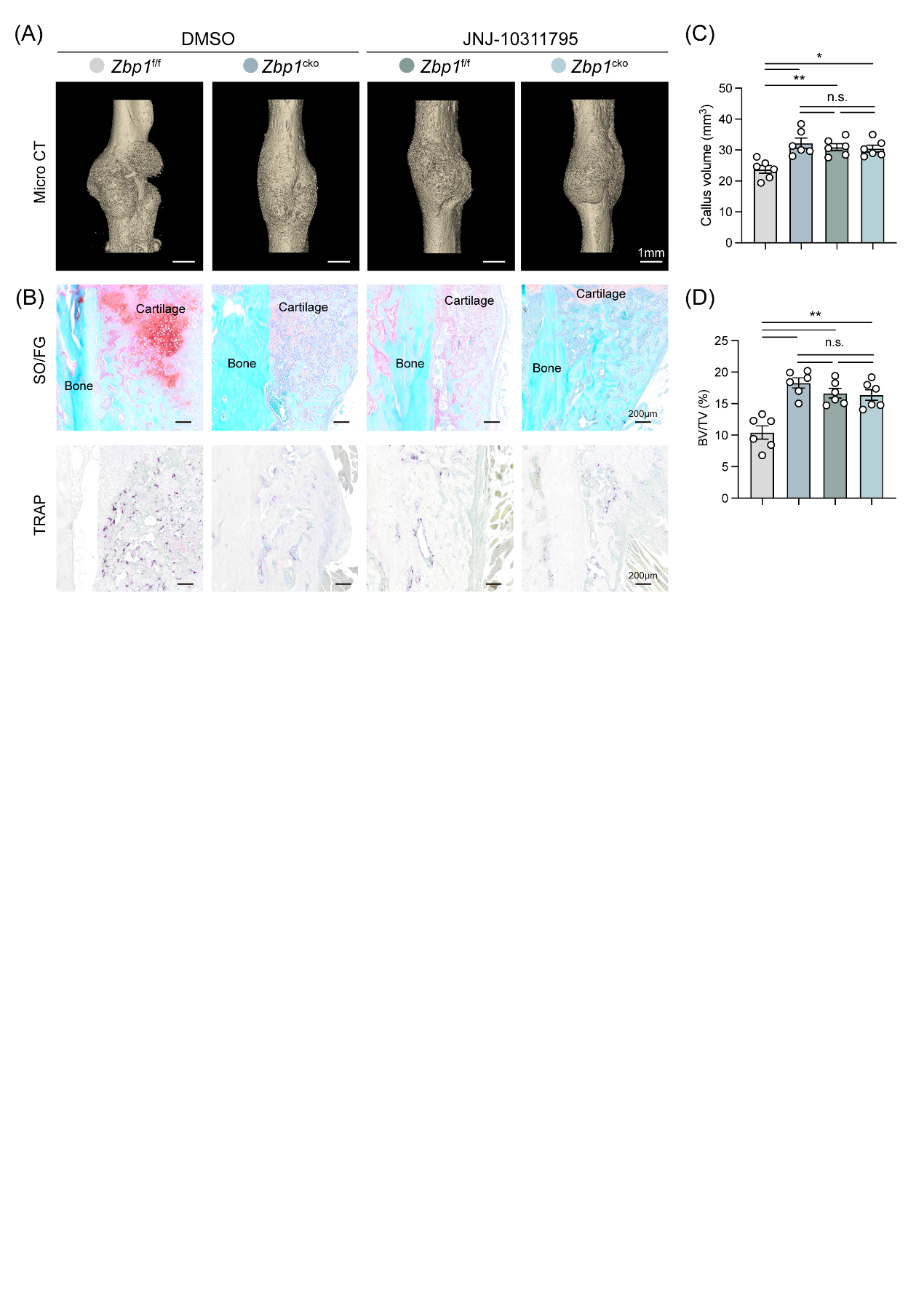


**Figure S9. JNJ-10311795 impairs bone fracture healing by targeting ZBP1 in macrophages.**

(A) Representative three-dimensional micro-CT reconstructions of femoral fracture calluses from *Zbp1*^f/f^ and *Zbp1*^cko^ mice treated with DMSO or JNJ-10311795 at day 14 post-fracture. Scale bar, 1 mm. (B) Representative safranin O/fast green (SO/FG) and TRAP-stained sections of the femoral fracture callus in the indicated groups. Scale bar, 200 μm. (C) Quantification of callus volume in the indicated groups (*n*=6). JNJ-10311795 significantly reduced callus volume in *Zbp1*^f/f^ mice, but this effect was abolished in *Zbp1*^cko^ mice. (D) Micro-CT analysis of fracture callus microarchitecture (BV/TV) in the indicated groups (*n*=6). JNJ-10311795 significantly decreased BV/TV in *Zbp1*^f/f^ mice, but had no significant effect in *Zbp1*^cko^ mice. All data are presented as mean ± SD (n.s., no significance, **P* < 0.05, ***P* < 0.01).

**Supplemental Tables**

**Table S1. Primer sequences for mouse genotyping by PCR**

| Target Gene | Primer Name | Sequence (5’ to 3’) | Product Size |
| --- | --- | --- | --- |
| *Piezo1* | Piezo1-F1 | GGGACAATGAGGTAGGATTGATG | WT: 324 bp |
|  | Piezo1-R1 | AGACAAGGTTCTGGGACCCTATG | FL: 429 bp |
| *Zbp1* | Zbp1-F1 | GGAGGATTGCTATGAGTTCCAGG | WT: 288 bp  FL: 392 bp |
|  | Zbp1-R1 | CCTGATACAGCAGGAGTCCTGAA |  |
| *Cx3cr1*(Cre) | Cx3cr1-Cre-F1 | CGTGATCTGGTTTGCTGCATACAG | Ki: 368 bp |
|  | Cx3cr1-Cre-R1 | CAGCAGGGAACCATTTCCTGTTGTT |  |
|  | Cx3cr1-Cre-F2 | CGTGATCTGGTTTGCTGCATACAG | WT: 258 bp |
|  | Cx3cr1-Cre-R2 | AAGACGGACAGGAAGATGGTTCCA |  |

**Table S2. PCR reaction mixture for genotyping**

| Components | Volume per Reaction (μL) |
| --- | --- |
| 2×Taq Master Mix（Vazyme P112-03） | 12.5 |
| ddH_2_O | 9.5 |
| Primer F（10 μM） | 1 |
| Primer R（10 μM） | 1 |
| Template (≈100 ng/μL) | 1 |

**Table S3. Thermal cycling protocol for genotyping PCR**

| Temperature | Time | Cycles |
| --- | --- | --- |
| 95℃ | 5 min | 1 |
| 98℃ | 30 s | 20 |
| 65℃（-0.5℃/cycle） | 30 s |  |
| 72℃ | 45 s |  |
| 98℃ | 30 s | 20 |
| 55℃ | 30 s |  |
| 72℃ | 45 s |  |
| 72℃ | 5 min | 1 |
| 10℃ | ∞ |  |

**Table S4. Ranking of candidate compounds based on Gibbs free energy values**

| DB04016 | 0.97367558 | DB15399 | 0.16995001 | DB02555 | 0.10497238 |
| --- | --- | --- | --- | --- | --- |
| DB06435 | 0.47588843 | DB12411 | 0.1682243 | DB11830 | 0.0989011 |
| DB08487 | 0.32187712 | DB07827 | 0.16009281 | DB08901 | 0.07874865 |
| DB01897 | 0.28205128 | DB00210 | 0.15740741 | DB12345 | 0.06837607 |
| DB03642 | 0.2755102 | DB15328 | 0.15207373 | DB00984 | 0.06382979 |
| DB07691 | 0.21359223 | DB02449 | 0.14285714 | DB11791 | 0.06157113 |
| DB02473 | 0.21212121 | DB15382 | 0.13250283 | DB15345 | 0.05708245 |
| DB01003 | 0.20918984 | DB05039 | 0.13122172 | DB03268 | 0.05152471 |
| DB03336 | 0.20048019 | DB03466 | 0.12866817 | DB06638 | 0.04931794 |
| DB03072 | 0.18483412 | DB05984 | 0.12485939 | DB12673 | 0.0373444 |
| DB12690 | 0.17508813 | DB13520 | 0.1148272 | DB03038 | 0.02986612 |
| DB12561 | 0.17233294 | DB15133 | 0.10619469 | All remaining | 0 |

**Table S5. Primer sequences for qPCR analysis**

| Gene Name | Species | Sequence (5’ to 3’) | |
| --- | --- | --- | --- |
| *GAPDH* | Human | F: | CTCCTGCACCACCAACTGCT |
|  |  | R: | GGGCCATCCACAGTCTTCTG |
| *PIEZO1* | Human | F: | CTCTTCCTGGCGCTGTTC |
|  |  | R: | GATGAGGTTGGTGGAGTTGG |
| *IL1B* | Human | F: | TGGGATCCTCTCCAGCCAAGC |
|  |  | R: | AGCCCTTCATCTTTTGGGGTCCG |
| *TNF* | Human | F: | CCACTTCGAAACCTGGGATTC |
|  |  | R: | TTAGTGGTTGCCAGCACTTCA |
| *Gapdh* | Mouse | F: | AGGTCGGTGTGAACGGATTTG |
|  |  | R: | TGTAGACCATGTAGTTGAGGTCA |
| *Piezo1* | Mouse | F: | CTTACACGGTTGCTGGTTGG |
|  |  | R: | CACTTGATGAGGGCGGAAT |
| *Zbp1* | Mouse | F: | AAGAGTCCCCTGCGATTATTTG |
|  |  | R: | TCTGGATGGCGTTTGAATTGG |
| *Il-1β* | Mouse | F: | TCCAGGATGAGGACATGAGCAC |
|  |  | R: | GAACGTCACACACCAGCAGGTTA |
| *Il-6* | Mouse | F: | CCACTTCACAAGTCGGAGGCTTA |
|  |  | R: | GCAAGTGCATCATCGTTGTTCATAC |
| *Tnf-α* | Mouse | F: | GTTCTATGGCCCAGACCCTCAC |
|  |  | R: | GGCACCACTAGTTGGTTGTCTTTG |
| *Nfatc1* | Mouse | F: | CACATTCTGGTCCATACGA |
|  |  | R: | CGTGTAGCTGCACAATGG |
| *Dcstamp* | Mouse | F: | TGGAAGTTCACTTGAAACTACGTG |
|  |  | R: | CTCGGTTTCCCGTCAGCCTCTCTC |
| *Ctsk* | Mouse | F: | CAGCTTCCCCAAGATGTGAT |
|  |  | R: | GAAGCACCAACGAGAGGAGA |
| *Atp6v0d2* | Mouse | F: | CAGAGCTGTACTTCAATGTGGAC |
|  |  | R: | AGGTCTCACACTGCACTAGGT |
| *Acp5* | Mouse | F: | TCCTGGCTCAAAAAGCAGTT |
|  |  | R: | ACATAGCCCACACCGTTCTC |
| *Trap* | Mouse | F: | CAGCAGCCAAGGAGGACTAC |
|  |  | R: | ACATAGCCCACACCGTTCTC |

**Table S6. qPCR reaction mixture**

| Components | Volume per Reaction (μL) |
| --- | --- |
| SYBR PCR Master Mix | 5 |
| Primer F（10 μM） | 0.4 |
| Primer R（10 μM） | 0.4 |
| cDNA | 2 |
| ddH_2_O | 2.2 |

**Table S7. Thermal cycling protocol for qPCR**

| Temperature | Time | Cycles |
| --- | --- | --- |
| 95℃ | 30 s | 1 |
| 95℃ | 10 s | 40 |
| 60℃ | 30 s |  |
| 95℃ | 15 s |  |
| 60℃ | 60 s | 1 |
| 95℃ | 15 s |  |

**Table S8. Human subject clinical characteristics**

| **Variable** | **qPCR (7d)** | **qPCR (14d)** | **IF** |
| --- | --- | --- | --- |
| **n** | 6 | 6 | 6 |
| **Corresponding figures** | Fig. 1C-E  Fig. 3H, I | Fig. 1C-E  Fig. 3H, I | Fig. 1F, G |
| **Age (years, mean ± SD)** | 16.8 ± 3.8 | 17.7 ± 4.4 | 17.7 ± 3.9 |
| **Sex, n (%)** |  |  |  |
| Male | 3 (50.0%) | 3 (50.0%) | 3 (50.0%) |
| Female | 3 (50.0%) | 3 (50.0%) | 3 (50.0%) |
| **Angle classification, n (%)** |  |  |  |
| Class Ⅰ | 2 (33.3%) | 2 (33.3%) | 2 (33.3%) |
| Class Ⅱ | 4 (66.7%) | 4 (66.7%) | 4 (66.7%) |
| **Degree of crowding, n (%)** |  |  |  |
| Moderate | 2 (33.3%) | 2 (33.3%) | 2 (33.3%) |
| Severe | 4 (66.7%) | 4 (66.7%) | 4 (66.7%) |
| **Probing depth**  **(mm, mean ± SD)** | 2.12 ± 0.19 | 2.20 ± 0.18 | 2.23 ± 0.18 |
| **Orthodontic force** | 0.014-inch CuNiTi archwire | | |
