# Supplementary material for: Macrophage piezo1 senses mechanical force to drive osteoclastogenesis via ZBP1: Implications for bone remodelling therapy
Source: Clin Transl Med. 2026 May 23;16(5):e70703. doi: 10.1002/ctm2.70703 (PMC13239690; doi:10.1002/ctm2.70703)
Supplement: Supplementary file 3 — Supporting Information [file CTM2-16-e70703-s001.docx]

**Supplementary materials**

**Supplemental Figures**

**Figure S1**

**
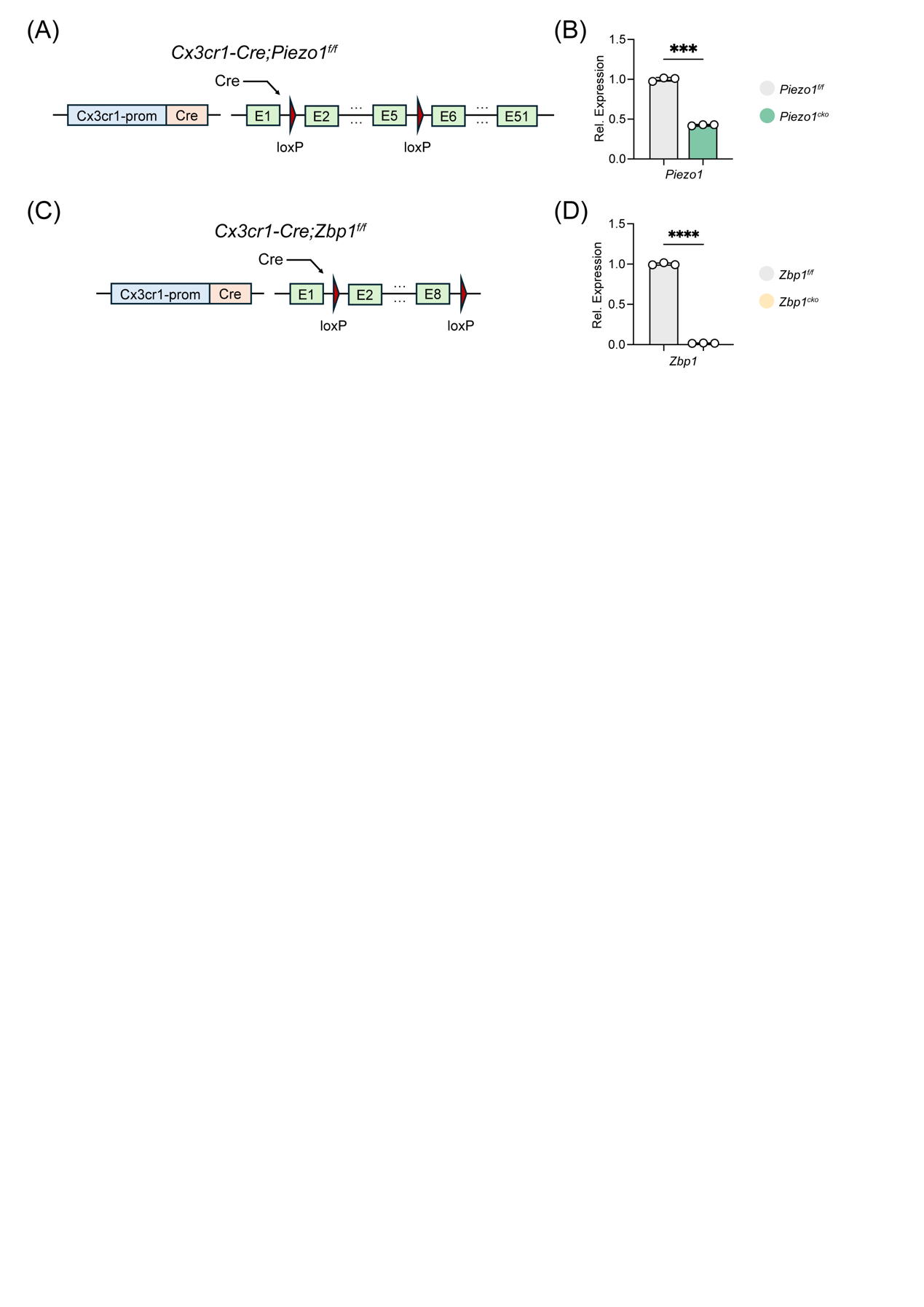
**

**Figure S1. Generation of conditional knockout mice.**

(A) Illustration of *Piezo1* deletion in *Cx3cr1*-expressing macrophages. (B) mRNA expression of *Piezo1* in BMDMs of *Piezo1^f/f^* and *Piezo1^cko^* mice was measured by qPCR (*n* = 3). (C) Illustration of *Zbp1* deletion in *Cx3cr1*-expressing macrophages. (D) mRNA expression of *Zbp1* in BMDMs of *Zbp1^f/f^* and *Zbp1^cko^* mice was measured by qPCR (*n* = 3).

**Figure S2**

**
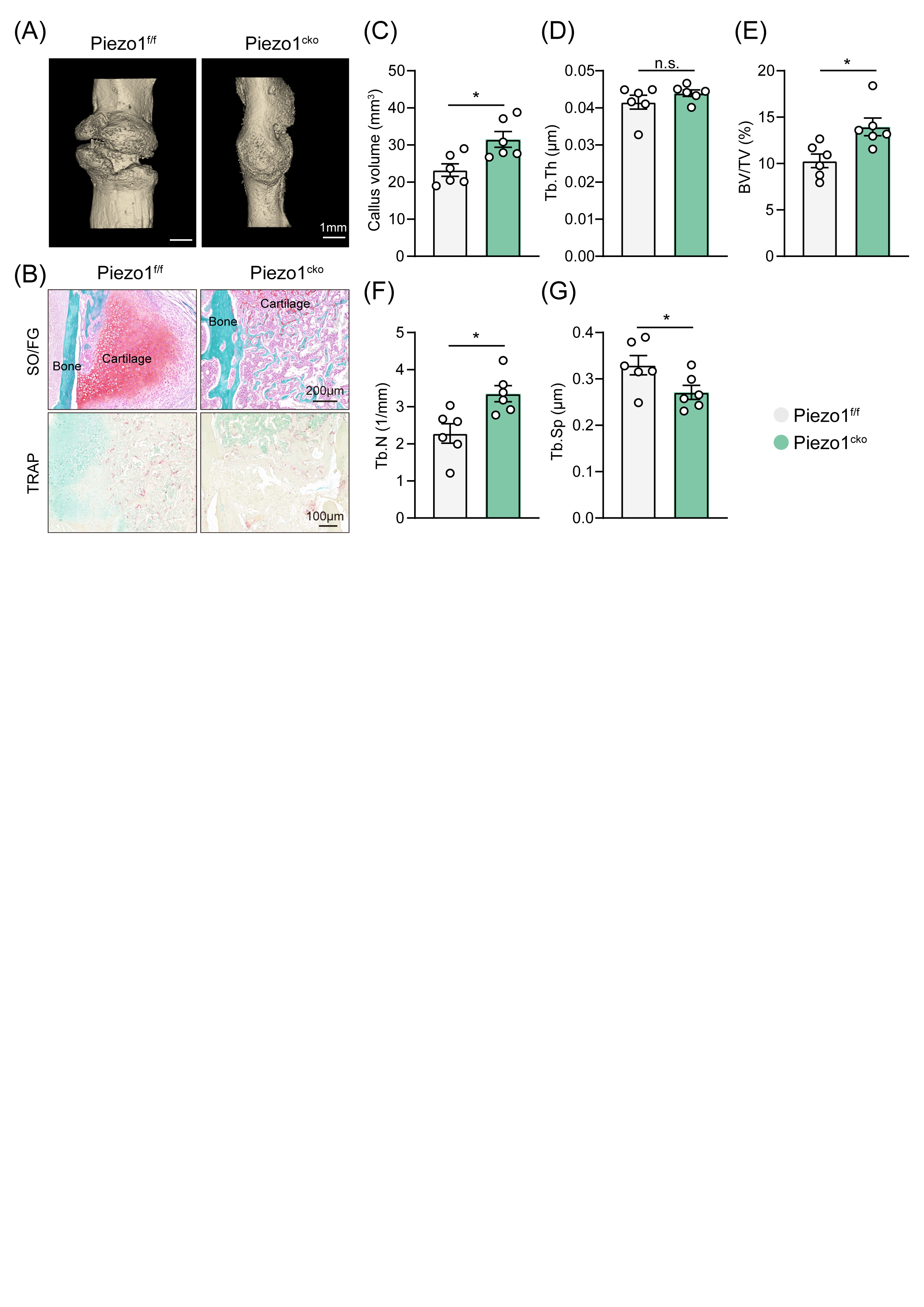
**

**Figure S2. Macrophage-specific *Piezo1* deletion alters fracture healing pattern.**

(A) Representative micro-CT reconstructions of femoral fracture callus in *Piezo1^f/f^* and *Piezo1^cko^* mice at the healing endpoint (*n*=6). (B) Representative safranin O/fast green- and TRAP-stained sections of the femoral fracture callus in *Piezo1^f/f^* and *Piezo1^cko^* mice. Scale bars of safranin O/fast green staining images, 200 μm; Scale bars of TRAP staining images, 100 μm. (C) Quantification of callus volume in *Piezo1^f/f^* and *Piezo1^cko^* mice at the healing endpoint (*n*=6). (D-G) Micro-CT analysis of fracture callus microarchitecture, including Tb.Th, BV/TV, Tb.N, and Tb.Sp (*n*=6). All data are presented as mean ± SD (n.s., no significance, **P* < 0.05).

**Figure S3**

**
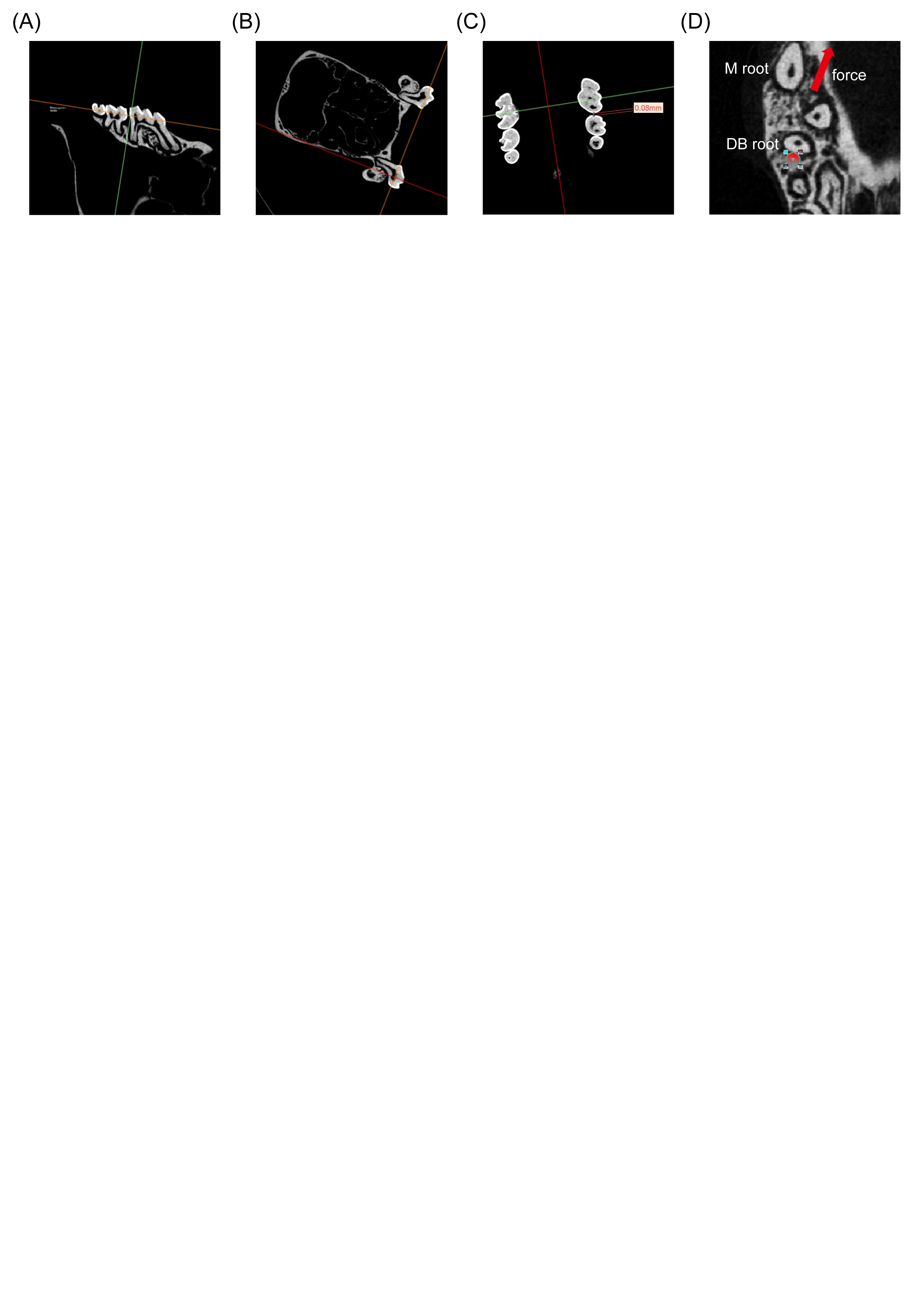
**

**Figure S3. Schematic diagram of Micro-CT measurement in mice.**

(A-C) Micro-CT measurement of OTM distance. ((A) sagittal plane, (B) coronal plane, (C) horizontal plane). (D) Micro-CT analysis of periradicular bone morphometry.

**Supplemental Tables**

**Table S1. Primer sequences for mouse genotyping by PCR**

| Target Gene | Primer Name | Sequence (5′ to 3′) | Product Size |
| --- | --- | --- | --- |
| *Piezo1* | Piezo1-F1 | GGGACAATGAGGTAGGATTGATG | WT: 324 bp |
|  | Piezo1-R1 | AGACAAGGTTCTGGGACCCTATG | FL: 429 bp |
| *Zbp1* | Zbp1-F1 | GGAGGATTGCTATGAGTTCCAGG | WT: 288 bp  FL: 392 bp |
|  | ZBP1-R1 | CCTGATACAGCAGGAGTCCTGAA |  |
| *Cx3cr1*(Cre) | Cx3cr1-Cre-F1 | CGTGATCTGGTTTGCTGCATACAG | Ki: 368 bp |
|  | Cx3cr1-Cre-R1 | CAGCAGGGAACCATTTCCTGTTGTT |  |
|  | Cx3cr1-Cre-F2 | CGTGATCTGGTTTGCTGCATACAG | WT: 258 bp |
|  | Cx3cr1-Cre-R2 | AAGACGGACAGGAAGATGGTTCCA |  |

**Table S2. PCR reaction mixture for genotyping**

| Components | Volume per Reaction (μl) |
| --- | --- |
| 2×Taq Master Mix（Vazyme P112-03） | 12.5 |
| ddH2O | 9.5 |
| Primer F（10μM） | 1 |
| Primer R（10μM） | 1 |
| Template (≈100ng/μl) | 1 |

**Table S3. Thermal cycling protocol for genotyping PCR**

| Temperature | Time | Cycles |
| --- | --- | --- |
| 95℃ | 5min | 1 |
| 98℃ | 30s | 20 |
| 65℃（-0.5℃/cycle） | 30s |  |
| 72℃ | 45s |  |
| 98℃ | 30s | 20 |
| 55℃ | 30s |  |
| 72℃ | 45s |  |
| 72℃ | 5min | 1 |
| 10℃ | ∞ |  |

**Table S4. Ranking of candidate compounds based on Gibbs free energy values**

| DB04016 | 0.97367558 | DB15399 | 0.16995001 | DB02555 | 0.10497238 |
| --- | --- | --- | --- | --- | --- |
| DB06435 | 0.47588843 | DB12411 | 0.1682243 | DB11830 | 0.0989011 |
| DB08487 | 0.32187712 | DB07827 | 0.16009281 | DB08901 | 0.07874865 |
| DB01897 | 0.28205128 | DB00210 | 0.15740741 | DB12345 | 0.06837607 |
| DB03642 | 0.2755102 | DB15328 | 0.15207373 | DB00984 | 0.06382979 |
| DB07691 | 0.21359223 | DB02449 | 0.14285714 | DB11791 | 0.06157113 |
| DB02473 | 0.21212121 | DB15382 | 0.13250283 | DB15345 | 0.05708245 |
| DB01003 | 0.20918984 | DB05039 | 0.13122172 | DB03268 | 0.05152471 |
| DB03336 | 0.20048019 | DB03466 | 0.12866817 | DB06638 | 0.04931794 |
| DB03072 | 0.18483412 | DB05984 | 0.12485939 | DB12673 | 0.0373444 |
| DB12690 | 0.17508813 | DB13520 | 0.1148272 | DB03038 | 0.02986612 |
| DB12561 | 0.17233294 | DB15133 | 0.10619469 | All remaining | 0 |

**Table S5. Primer sequences for qPCR analysis**

| Gene Name | Species | Sequence (5′ to 3′) | |
| --- | --- | --- | --- |
| *GAPDH* | Human | F: | CTCCTGCACCACCAACTGCT |
|  |  | R: | GGGCCATCCACAGTCTTCTG |
| *PIEZO1* | Human | F: | CTCTTCCTGGCGCTGTTC |
|  |  | R: | GATGAGGTTGGTGGAGTTGG |
| *IL1B* | Human | F: | TGGGATCCTCTCCAGCCAAGC |
|  |  | R: | AGCCCTTCATCTTTTGGGGTCCG |
| *TNF* | Human | F: | CCACTTCGAAACCTGGGATTC |
|  |  | R: | TTAGTGGTTGCCAGCACTTCA |
| *Gapdh* | Mouse | F: | AGGTCGGTGTGAACGGATTTG |
|  |  | R: | TGTAGACCATGTAGTTGAGGTCA |
| *Piezo1* | Mouse | F: | CTTACACGGTTGCTGGTTGG |
|  |  | R: | CACTTGATGAGGGCGGAAT |
| *Zbp1* | Mouse | F: | AAGAGTCCCCTGCGATTATTTG |
|  |  | R: | TCTGGATGGCGTTTGAATTGG |
| *Il-1β* | Mouse | F: | TCCAGGATGAGGACATGAGCAC |
|  |  | R: | GAACGTCACACACCAGCAGGTTA |
| *Il-6* | Mouse | F: | CCACTTCACAAGTCGGAGGCTTA |
|  |  | R: | GCAAGTGCATCATCGTTGTTCATAC |
| *Tnf-α* | Mouse | F: | GTTCTATGGCCCAGACCCTCAC |
|  |  | R: | GGCACCACTAGTTGGTTGTCTTTG |

**Table S6. qPCR reaction mixture**

| Components | Volume per Reaction (μl) |
| --- | --- |
| SYBR PCR Master Mix | 5 |
| Primer F（10μM） | 0.4 |
| Primer R（10μM） | 0.4 |
| cDNA | 2 |
| ddH2O | 2.2 |

**Table S7. Thermal cycling protocol for qPCR**

| Temperature | Time | Cycles |
| --- | --- | --- |
| 95℃ | 30s | 1 |
| 95℃ | 10s | 40 |
| 60℃ | 30s |  |
| 95℃ | 15s |  |
| 60℃ | 60s | 1 |
| 95℃ | 15s |  |
